# An Overview of Microorganisms Immobilized in a Gel Structure for the Production of Precursors, Antibiotics, and Valuable Products

**DOI:** 10.3390/gels10100646

**Published:** 2024-10-10

**Authors:** Dmitriy Berillo, Turganova Malika, Baiken B. Baimakhanova, Amankeldi K. Sadanov, Vladimir E. Berezin, Lyudmila P. Trenozhnikova, Gul B. Baimakhanova, Alma A. Amangeldi, Bakhytzhan Kerimzhanova

**Affiliations:** 1 LLP “Research and Production Center for Microbiology and Virology”, Almaty 050000, Kazakhstan; bbbayken@mail.ru (B.B.B.);; 2 Department of Chemistry and Biochemical Engineering, Satbayev University, Almaty 050013, Kazakhstan; malika.turganova@stud.satbayev.university; 3 Department of Pharmaceutical and Toxicological Chemistry, School of Pharmacy, Asfendiyarov Kazakh National Medical University, Almaty 050000, Kazakhstan; 4 JSC “Scientific Center of Anti-Infective Drugs”, Almaty 050000, Kazakhstan

**Keywords:** bacteria, immobilization, enzyme, fungi, pharmaceutical, biotechnology, microbiology, precursor

## Abstract

Using free microorganisms for industrial processes has some limitations, such as the extensive consumption of substrates for growth, significant sensitivity to the microenvironment, and the necessity of separation from the product and, therefore, the cyclic process. It is widely acknowledged that confining or immobilizing cells in a matrix or support structure enhances enzyme stability, facilitates recycling, enhances rheological resilience, lowers bioprocess costs, and serves as a fundamental prerequisite for large-scale applications. This report summarizes the various cell immobilization methods, including several synthetic (polyvinylalcohol, polyethylenimine, polyacrylates, and Eudragit) and natural (gelatin, chitosan, alginate, cellulose, agar–agar, carboxymethylcellulose, and other polysaccharides) polymeric materials in the form of thin films, hydrogels, and cryogels. Advancements in the production of well-known antibiotics like penicillin and cephalosporin by various strains were discussed. Additionally, we highlighted cutting-edge research related to strain producers of peptide-based antibiotics (polymyxin B, Subtilin, Tyrothricin, varigomycin, gramicidin S, friulimicin, and bacteriocin), glusoseamines, and polyene derivatives. Crosslinking agents, especially covalent linkers, significantly affect the activity and stability of biocatalysts (penicillin G acylase, penicillinase, deacetoxycephalosporinase, L-asparaginase, β-glucosidase, Xylanase, and urease). The molecular weight of polymers is an important parameter influencing oxygen and nutrient diffusion, the kinetics of hydrogel formation, rigidity, rheology, elastic moduli, and other mechanical properties crucial for long-term utilization. A comparison of stability and enzymatic activity between immobilized enzymes and their free native counterparts was explored. The discussion was not limited to recent advancements in the biopharmaceutical field, such as microorganism or enzyme immobilization, but also extended to methods used in sensor and biosensor applications. In this study, we present data on the advantages of cell and enzyme immobilization over microorganism (bacteria and fungi) suspension states to produce various bioproducts and metabolites—such as antibiotics, enzymes, and precursors—and determine the efficiency of immobilization processes and the optimal conditions and process parameters to maximize the yield of the target products.

## 1. Introduction

In 2023, the worldwide market of biopharma microbiology reached USD 6 billion, and it is forecasted to grow to USD 18 billion by 2036 [1]. Penicillin G acylase is a main industrial enzyme that is used in the enzymatic production of 20,000 t/year of 6-aminopenicillanic acid, which is the industrial β-lactam intermediate used for chemical modification. The global market of penicillin G acylase reached approximately USD 672 million in 2022 and might reach USD 1100 million in 2031 [2].

Vandamme, in their 1983 review, highlighted various methods of cell-based bioreactors for the conversion of peptide antibiotics into therapeutically useful active pharmaceutical ingredients, as well as the utilization of free solution and encapsulated penicillin acylases for the production of the penicillin nucleus 6-aminopenicillanic acid and 7-aminocephalosporanic acid, in addition to its following transformation to numerous semisynthetic β-lactamic antibiotics (penicillins and cephalosporins) [3].

One of the first reports of the immobilization of spores of *Streptomyces aureofaciens* ATCC 10762 in calcium alginate gel was described in 1987, leading to the production of the widely used chlortetracycline [4]. Later, Barbotin’s group established that carrageenan and potassium chloride concentrations significantly affect the metabolic pathway of *S. aureofaciens* and change the yield of chlortetracycline and tetracycline. The immobilized strain revealed the tetracycline release more than eight times (12.3 mg/g) the biomass for immobilized cells compared to the (1.5 mg/g) biomass for free cells [5].

Nowadays, changing the culturing medium and immobilization strategy, as well as gene manipulation, can result in a better product yield. Thus, chlortetracycline titers were increased to 2.15 and 3.3 g/L, respectively, in the engineering strains SRC2 and SRC3 with improved *ctcB* expression. Briefly, two cluster-situated resistance genes were co-overexpressed with *ctcB*, which creates up to 3.8 g/L of strain-produced chlortetracycline in dynamic fermentation, illustrating a 38-fold increase compared to the original strain [6]. A novel biocatalyst was engineered from the wild-type penicillin acylase of *Escherichia coli* by introducing mutations at positions α146 and β24 (βF24A/αF146Y) and covalently attached on a modified industrial commercially available support (glyoxyl eupergit C250L). The engineered biocatalyst achieved a faster conversion speed in the synthesis of cephalexin (99% vs. 76%), cefaclor (99% vs. 65%), and cefprozil (99% vs. 60%) compared to the wild-type enzyme. The production of cephalexin utilizing the immobilized βF24A/αF146Y acylase was scaled up to 30 L with a sustainable average yield of 93% after 20 cycles [7].

The review by Sheldon (2005) summarizes research reports related to enzyme immobilization on organic and inorganic carriers [8]. All process parameters are crucial to consider during microorganism immobilization. As noted by Mussenden et al., the increase in the content of the immobilized spore (4 × 10^4^ to 2000 × 10^4^ spores /mL gel) and initial bead diameter (from 3.5–4.0 to 1.5–2.0 mm) led to an enhanced yield of the penicillin titer from 0.2 to 1.2 g/L [9]. The adsorption of biomolecules on the macropore surface improves the loading degree and diminishes the internal mass transfer resistance. Thus, penicillin G acylase’s immobilization level reached 895 mg/g (dry scaffold), and the apparent fermentative activity reached 1033 U/g (dry scaffold) [10].

Park and Khang discovered that cephalosporin C (CPC) production was primarily affected by the size of Ba-alginate beads, while the PEI concentration had less of an impact. Moreover, minimal production media had a positive effect and enhanced CPC productivity by 3.4 folds compared with chemically defined media employed in fed-batch fermentations. Immobilized cells in fed-batch fermentation can continuously generate CPC for a month, yielding approximately 7-fold more free microorganisms than in batch fermentation [11]. Kundu et al. utilized a green and sustainable whole-cell immobilization approach, which holds significant potential for CPC biosynthesis by maintaining large cell densities and reducing broth-handling issues. Given that CPC fermentation is highly aerobic, the symbiotic relationship between *Cephalosporium acremonium* and *Chlorella pyrenoidosa* was leveraged to enhance oxygen transfer to the fungi through co-immobilization with algae [12].

It is worth considering all methods of microorganism immobilization on various scaffolds used for environmental application in order to select a perspective-appropriate technique applicable for biopharmaceutical purposes.

Mehrotra et al. generalized data on various pollutants eliminated with the help of immobilized bacteria, and special attention was paid to various mechanisms using immobilized cells to restore the environment—for example, cells that are enclosed in a hydrogel and their participation in the neutralization of harmful pollutants and the maintenance of environmental rehabilitation [13].

Zur and co-authors presented a review of microorganisms living on natural surfaces, which contribute to the formation of biofilms. These microorganisms are described by the genetic and physiological diversity applied by the microenvironment inside the matrix. Such microbial cells present many metabolic variants compared with their free-living analogs. The process of the immobilization of bacteria can take place both naturally and artificially. Still, most of the changes observed in immobilized cells are associated with protective effects for which the use of auxiliary materials is necessary [14]. Immobilized cells contribute to an increase in the efficiency of removing pollutants from water by more than 60%. The key factors contributing to the design of immobilized cells are presented, beginning with the choice of a carrier for immobilization and methods of immobilization [15]. Next, immobilized bacterial gels were introduced, namely, their production and application. In addition, examples of bacterial-based fermentors created by straight crosslinking of bacteria with a minimum number of polymers were presented. Some focus is on environmental applications, i.e., bacterial bioreactors illustrated promising data for removing phenolic compounds (pesticides and pharmaceutical wastewater treatment) [16].

In a book chapter devoted mainly to cryogels, macroporous hydrogels were covered, including composites with viable microorganisms used to create scaffolds and supports for immobilizing cells such as bacteria and viruses. Cryogels are macroporous polymer gels formed at sub-zero temperatures by a process known as cryogel formation. Activated carbon and inorganic minerals (clays, metal oxides, and zeolites) and agricultural wastes are satisfactory scaffolds for bacterial immobilization, too, but have problems associated with limiting biomass loading and leaching of bacteria in large-scale industrial processes [17]. Zeolites and some clays contain enormous surface and Si-OH functionalities that can be covalently modified with aminogroups or hydrophobic groups via treatment with corresponding triethoxysilane derivatives. These surfaces can be successfully used for efficient microorganism adsorption or covalent attachment. These carriers are robust, and it is unlikely that the leakage of unwanted compounds will occur over time. Evolutionally, these carriers are not harmful to selected strains of bacteria. Synthetic and natural polymers with various functionalities are utilized to effectively immobilize microorganisms and cells acceptable for following cultivation in sterile conditions. Synthetic polymeric carriers have a carbon–carbon backbone, and therefore, these materials may be prepared with given mechanical properties and required porosity characteristics. There is no substrate/product diffusion restriction in the case of synthetic scaffolds due to the monolayer of enzymes, while embedded biomolecules, mostly balk natural polysaccharides, have disadvantages [18].

The study of bioremediation with immobilization technology for microorganisms contributes to treating wastewater contaminated with microcystines and variational types of additional materials and methods for treating such contaminated water [19]. Incorporating cells into various scaffolds is widely used in biocatalytic research; for example, it includes food and dairy manufacturing and synthesizing valuable goods such as biopharmaceuticals, bioplastics, additives, and biofuels. In addition, this area is actively developing in bioremediation, the creation of biosensors, and tissue regeneration in medicine [20].

Bacterial spores are cost-effective to produce and exhibit high resistance to environmental stresses. The spore’s core is surrounded by several layers, including the inner membrane, germ cell wall, cortex, outer membrane, spore coat, and, in certain species, the exosporium. The exterior of the spore is distinguished by an abundance of anions, hydrophobicity, and a variety of functional groups that can interact with and stabilize enzyme molecules through electrostatic forces, hydrophobic interconnection, and covalent bonding. Due to their probiotic properties derived from non-toxic bacterial strains, spores are ideal carriers for biocatalyst immobilization, particularly for food-grade enzymes or those intended for therapeutic applications. The incorporation of spores can occur via straight adsorption, covalent attachment, or surface display during the sporulation phase [21].

Most recent reviews are devoted to solving environmental issues with the help of immobilized bacteria and fungi. As one can see, there is a gap in summarizing the advances in incorporating microorganisms in appropriate scaffolds for the production of drugs and pharmaceuticals. Rhizoctycin is a phosphate-containing oligopeptide antibiotic synthesized by the Gram-positive bacterium *B. subtilis* with an MIC of 0.016–256 µg/mL [22].

## 2. Overview of Microbially Produced Antibiotics and Their Chemical Structures

In Table 1, we summarize the structures of well-known antibiotics and highlight the following groups: condensed hererocyclic derivatives such as penicillins, cephalosporins, and carbopenems; polypeptide-based structures: friulimicin, varigomycin, bacteriocin, bacitracin, polymyxins, etc.; four fused rings with a linear array of carbons—tetracyclines; steroidal ring-based structure fusidic acid, etc.; aminoglycosides—amino sugars linked by glycosidic bonds (Neomycin); and glycopeptides—a complex structure with sugar moieties and peptide-linked aromatic rings.

Scientists in Southeast Asia tracked the species of hazardous probiotic-producing substances in the intestines of buffaloes (*Bubalus bubalis*), isolating 12 strains from a variety of specimens, such as milk, rumen, and feces of Nilli Ravi buffaloes. The potential for antimicrobial activity had an inhibition degree of 0.28 cm against *S. typhimurium*, 0.23 cm for Listeria monocytogenes, and 21 mm for *E. coli*. The greatest resistance was observed for the NMCC-Ru2-tested agacinth antibiotic—25.5 mmol of tetracycline [23].

*Bacteria Actinoplanes friuliensis* produce the lipopeptide antibiotic friulimicin. Friulimicin has a structure similar to daptomycin and has high antibacterial activity at MIC concentrations for *Streptomyces lividans.* When TK23-transformed with the control vector, pEM4 was 1 to 5 μg/mL, while when transformed with pEM4ExpA, the MIC increased to 50 to 75 μg/mL [24]. Table 1 presents all the aforementioned structures of antibiotics to provide an idea regarding the functional groups and classes.

**Table 1:** The chemical structure of antibiotics produced in microorganisms.

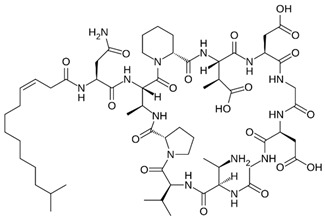 Friulimicin, cyclicpeptide structure [25]	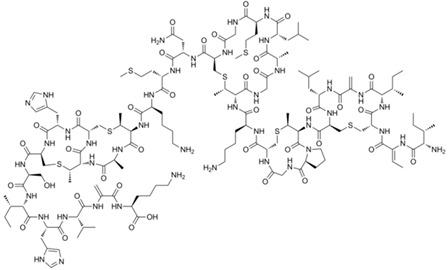 Bacteriocin, peptide structure [26]
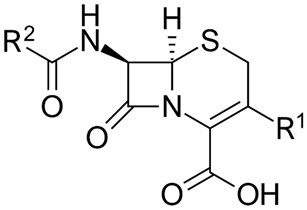 Core structure of the cephalosporins	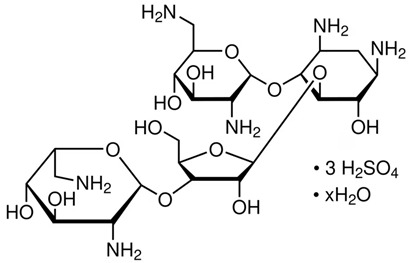 Neomycin glycosideamine [27,28] https://pubchem.ncbi.nlm.nih.gov/compound/Neomycin-C#section=Toxicity
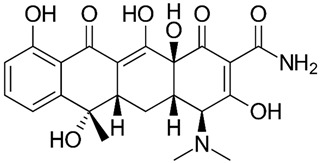 Tetracyclines [29,30]	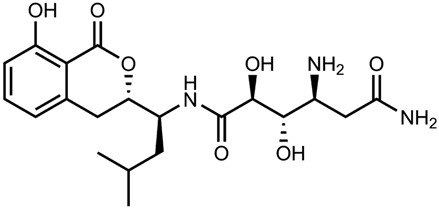 Amikumacin A [31]
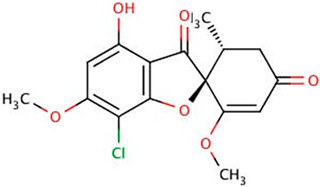 Griseofulvin [32]	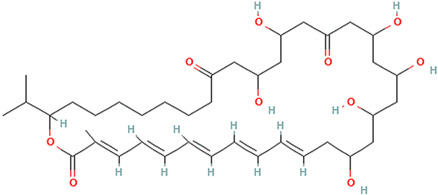 Roseofungin [33]
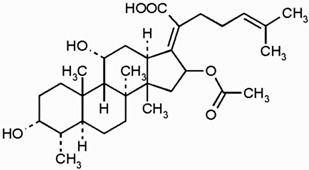 Fusidic acid [34]	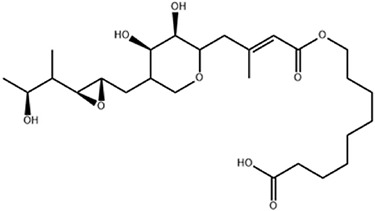 Mupirocin [35]
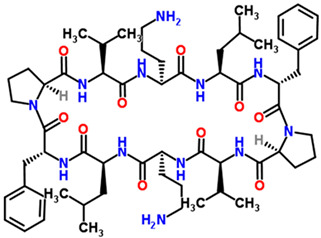 Gramicidin S cyclic peptide	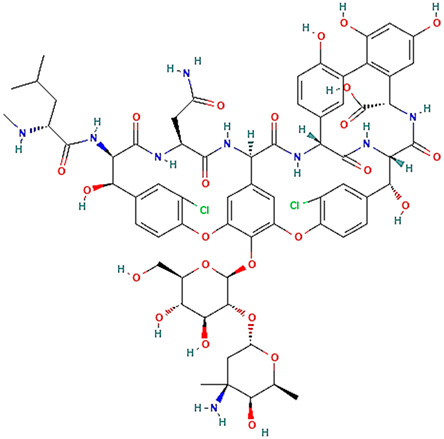 Varigomycin [36]
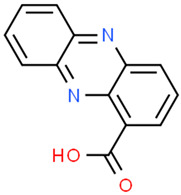 Tubermycin B	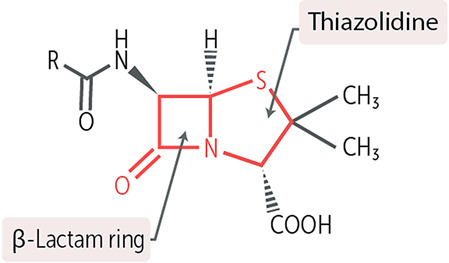 Penicillins
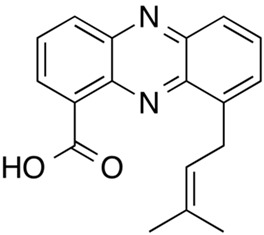 Endophenazine A	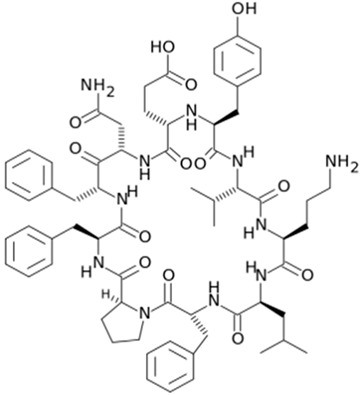 Tyrothricin cyclic peptide structure
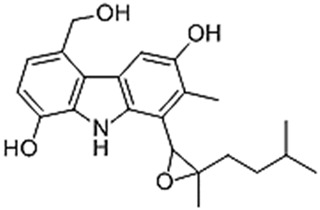 EpoCarbazolin B [37]	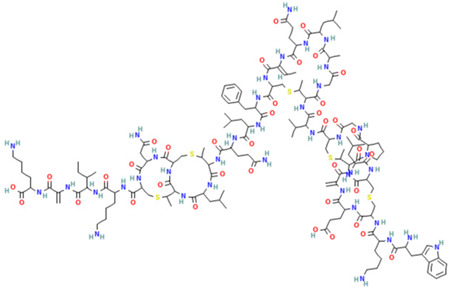 Subtilin peptide based structure [38]
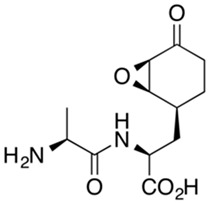 Bacilysin [39]	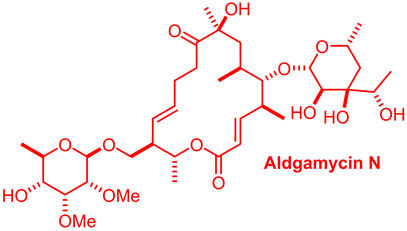 Aldgamycin N [40,41] 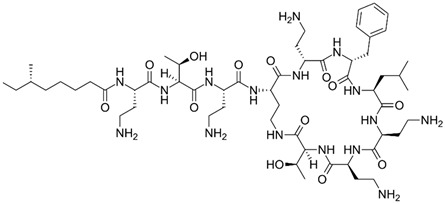 Polymyxin B [42]

Fosfomycin is a secondary metabolite isolated from *Streptomyces fradiae*; it has activity against *Staphylococcus aureus*, *Pneumococcus*, and Gram-negative bacteria MIC at ¼ 1 mg/L. Neomycin is an aminoglycoside antibiotic isolated from *Streptomyces fradiae* cultures MIC50/MIC90 at 8/256 µg/mL [43]. Tyrothricin is highly toxic to blood, the liver, kidneys, meninges, and the olfactory apparatus; therefore, it may only be utilized topically [44].

Amikumacin A has effectivity against 22 collective strains and 24 clinical isolates of bacteria and fungi. It was investigated, and researchers found substantial efficiency in multidrug-resistant clinical isolates *L. mesenteroides* VKPM B-4177 (V *S. aureus*, *S. epidermidis*, *C. krusei*, *Cr. neoformis*, and *Prototheca* spp. [45].

Roseofungin was originally produced from IMV A-23/791 of Streptomyces roseoflavus. The pentane macrocyclic derivative inhibits the viability of numerous phytopathogenic fungi, dermatophytes, and yeast-like fungi [36,46]. Tubermycin B (phenazine-1-carboxylic acid) is produced by *Pseudomonas 2HS*. Keudell et al. found that this strain secreted tiny amounts of a greenish-yellow pigment if cultured aerobically in a 1% yeast extract medium at 30 C and in the dynamic mode at 250 rpm. Interestingly, the microorganism synthesized more greenish-yellow pigment (2.16 mg/15 mL of culturing medium) if cultured in 0.5% 12-hydroxyoctadecanoic acid [36].

Varigomycin is a glycopeptide produced by Streptomyces orientalis. The mechanism of action of active pharmaceutical ingredients inhibits a specific step in synthesizing the peptidoglycan layer in the Gram-positive bacteria Staphylococcus aureus and Clostridium difficile [47]. The mbtH-like gene serves as a promising target for genetic engineering to enhance nonribosomal peptides production, as demonstrated by a 60% and 80% increase in vancomycin yield when overexpressing both cognate (Vcm11) and noncognate (CloY) MbtH-like proteins in *Amycolatopsis orientalis* KFCC10990P [48].

*Streptomyces anulatus*, *Bacillus megaterium* and *Bacillus subtilis* isolated from agricultural soils were assessed for antibiotic production and activity against seven bacteria tested. *Bacillus megaterium* was found to inhibit all pathogenic bacteria with prevalence rates of 12.9% and 18.8%, while *S. anulatus* was not active against *Proteus vulgaris*, and *B. subtilis* was inactive against *Enterococcus faecalis* [49].

*Bacillus lentus* (a producer of a bacteriocin-like substance), *Micrococcusroseus*, *Bacillus alvei* (Alvein), and *Bacillus pumillus* (amikumacin A) bacteria were isolated in the present study. The inhibitory activity of the isolated microorganisms was tested against some important opportunistic microflora, such as *Staphylococcus aureus* and *Pseudomonas* species [50]. *Bacillus lentus* is an available variant of subtilisin and served as a source of several subtilisin-like proteases, with an MIC of 5.4 mg/l. Parameters of culturing of 30 min, 60 °C, and pH 12 resulted in a >10^3^-fold decrease in infectivity [51].

Mupirocin is an antibiotic from the class of monocarboxylic acids obtained via the biosynthesis of *Pseudomonas fluorescens*, with an MIC of 0.064–1024 µg/mL. It is used as an antibacterial agent against MRSA in soft tissue or skin infections and nasal decolonization. Biotechnological production takes place via submerged fermentation and product extraction [52]. Aldgamycins are 16-membered macrolide antibiotics possessing the branched-chain sugar D-aldgarose or decarboxylated D-aldgarose at the fifth carbon position with an MIC of 3.5 µg/mL [53].

*The Streptomyces avidinii* strain was isolated from a soil sample collected in Ikeda, Hokkaido, Japan. This organism was cultured at 27 °C for 48 h at pH 7.2, keeping it constant with the help of CaCO_3_ in a 300 kg fermenter containing 150 kg of medium aldhamycin G and aldhamycin F, respectively, in pure form [54].

The ability of *Lactobacillus lactis* isolated from soil, yogurt, and cheese to produce antibiotics was investigated and cultured on MRS agar at a pH from 5.5 to 6.5 at 37 °C. A total of 96 isolates were identified as LAB-lantibiotic: *Lactococcus Lactis* (Subtilin), *Lactobacillus Brevis*, *Lactobacillus Brevis*, *Lactobacilus Rhamnosus*, *Lactobacillus Plantarum*, *Lactobacillus pentosus*, *Enterococcus feacalis* (bacteriocin), and Staphylococcus Simulans. The cell-free supernatants of eight strains showed inhibitory activity; for instance, *Enterococcus faecium* illustrated activity against *Bacillus subtlis* and *Pseudomonas aureginosa*, while *Pediococcus Damnosus* had less bacteriocin activity against *Staphylococcus aureus* and *Bacillus subtilis* [55].

The antibiotics vatasemicins A and B were obtained from a seawater specimen excavated from Toyama Bay, Japan, and identified as Streptomyces sp. The antimicrobial activity of vatasemicins A and B was studied. The former illustrated weak antibacterial effectiveness against *Staphylococcus aureus* and *Bacillus subtilis* at 12.5 and 25 μg/mL, respectively, and high efficiency against Gram-negative bacteria Proteus mirabilis at a concentration of 0.39 μg/mL, but activity against *Escherichia coli* was not observed [56].

Ahmadu Bello University in Samaru used soil samples to isolate fungi: *Aspergillus niger* (Coumarin, Griseofulvin), *Aspergillus fumigatus* (Coumarin), *Penicillium* sp. (Penicillin), and *Fusarium* sp. (P15) (Fusidic acid), producing antibiotics using the plate distribution method and serial dilution technique on Saboro agar, and performed a sensitivity test using test pathogens. As a result, *Aspergillus niger* produced zones of inhibition of 9 mm, 5 mm, and 6 mm for *Staphylococcus aureus*, *Escherichia coli*, and *Klebsiella pneumoniae*, and *Aspergillus fumigatus* revealed zones of inhibition of 5 mm against *Escherichia coli* and *Klebsiella pneumoniae*. In contrast, *Penicillium* sp. gave a zone size of 10 mm, 7 mm, and 6 mm against the same species of pathogens [57].

The study of the antimicrobial potential of mushroom extracts identified *Aspergillus* species, which was isolated from solid waste and showed great antibacterial activity against all tested bacteria, with a zone of inhibition of 2.2 cm against *E. coli* and *P. aeroginosa*, 2.0 cm against *C. albicans*, and 1.8 cm against *B. subtilis* [58]. The following fungi were isolated from untreated wastewater in Karlsruhe (Germany): *Fusarium sporotrichioides Sherb*, *Penicillium funiculosum*, and *Trichoderma harzianum Rafai* in the effluent, and *Aspergillus flavus*, *A. repens*, *A. fumigatus Fresenius*, and *A. fischeri*. Next, a small quantity of fungal mycelium was sown on fresh nutrient agar and Sabouraud agar. As a result, within 2 days of incubation, the bacteria *E. coli*, *Pseudomonas*, and *Enterococci* were applied in cross-strokes to the fungal colonies on the cups. In comparison with *A. flavus* and *A. fumigatus*, *P. chrysogenum* and *A. repens* were more efficient in inhibiting *Enterococci* and *P. chrysogenum*, and *P. notatum* had the greatest inhibitory effect on bifidobacteria [59].

## 3. Immobilization of Fungi

The immobilization of *Aspergillus fumigatus* and *Alternaria tenuissima* mycelia was performed to increase their capacity to produce paclitaxel using five various retention media: calcium alginate (Alg), agar–agar, Na-CMC, gelatin, and Arabian gum. Na-CMC was mixed with a mycelia fungi suspension and then added drop by drop to a FeCl_3_ solution for 1 h to complete the ion-exchange process and ionic crosslinking of beads. The productivity of paclitaxel was 558 ± 25 μg/L and 259 ± 13 μg/L in the first and second strains, respectively. Calcium alginate gel globules proved to be the most conductive and suitable retention carrier to maximize production. As a result, the impregnated mycelia of the respective mutants (694 and 388 μg/L) were determined to be promising in terms of obtaining the product. At the same time, immobilized agar–agar indicated moderate paclitaxel generation efficiency of 568 ± 15 μg/L and 279 ± 15 μg/L for the first and second strains, respectively [60].

Biosurfactant-based hydrophobization of polyvinyl alcohol (PVA) cryogels was used to immobilize hydrocarbon-oxidizing bacteria. The obtained hydrophobized 5 μL pellets of PVA cryogel contained sufficient viable bacterial cells per pellet (6500). As a result, using the immobilized biocatalyst, the oxidation efficiency of n-hexadecane was 51% after a 10-day incubation. Accordingly, PVA cryogels with enhanced hydrophobicity can incorporate bacterial cultures when performing oxidative alterations of organic compounds immiscible with water [61].

The immobilization of penicillin G acylase was performed on magnetic Ni, Mg, Zn, and Fe_3_O_4_ nanoparticles, which were arranged using the quick combustion method and calcination at 400 °C for 2 h. The surface of the nanoparticles was modified with sodium silicate hydrate, and PGA was crosslinked to the carrier particles using glutaraldehyde (GA). The activity of immobilized PGA reached 7121 U/g, the optimum pH for immobilized PGA was 8, the temperature was 45 °C, and immobilized PGA revealed higher stability to pH and temperature changes [62].

The metallo-beta-lactamase enzyme IMP-1, capable of hydrolyzing beta-lactam antibiotics, was immobilized on CNBr-activated Sepharose. The optimal temperatures of free enzyme activity were 70 °C, and that of immobilized enzymes was 60 °C. The optimal pH, 7.5, was changed to 6.5 after immobilization. Hence, the immobilization of IMP-1 by binding on carriers, the quantity of biocatalyst that can cause hydrolysis of 1 μmol of Pen G per minute, is defined as one unit of enzyme activity [63].

The screening of immobilized amoxicillin synthase was performed by adopting an amino-epoxide type carrier to produce an immobilized amoxicillin LK218 enzyme. The process includes adding incorporated amoxicillin enzyme LK218, 6-aminophenylacetic acid, and D-p-hydroxyphenylglycine to water; adjusting the pH of the mixture using HCl and NaOH solution; monitoring the temperature and reaction time of the mixture; and completing the reaction until the remaining concentration reaches 6-APA 0–2 mg/mL [64].

Using immobilized enzyme reactors involved integrated capillary electrophoresis (CE-IMER), which is performed based on Alg hydrogelization. Homogeneously encapsulated penicillinase comprised about 61.8% of the initial penicillinase in a mixture of sols encapsulated in a hydrogel matrix “egg carton”. The role of penicillinase is to selectively “recognize” and create the selective hydrolysis of penicillin G to form penicillinic acid [65].

This passage discusses the immobilization of penicillinase, a type of β-lactamase enzyme, into metal-organic frameworks (MOFs), specifically zeolitic imidazolate framework-8 (ZIF-8), using a self-assembly method. The study focuses on the catalytic performance of the β-lactamase@ZIF-8 porous material for the degradation of penicillins. Results show that β-lactamase@ZIF-8 demonstrated remarkable stability under harsh denaturing conditions, such as high temperatures, organic solvents, and exposure to enzyme inhibitors [66].

The immobilization of yeast involved *Debaryomyces hansenii*, *Pichia sorbitophila*, *Saccharomyces cerevisiae*, *Yarrowia lipolytica*, and *Zygosaccharomyces rouxii* in PVA hydrogel units utilizing the LentiKats process that were stored in sterile water at 4 °C, improving the survival rate of all species. The embedding of yeast in PVA-based hydrogel provides extended and inexpensive storage with very good cell viability and productivity, which is a favorable approach for industrial uses [67].

## 4. Immobilization of Actinomycetes

The immobilization of *Streptomyces clavuligerus* to produce antibiotics was crosslinked in the presence of viable cells using dialdehydes: glyoxal, glutardialdehyde, and modified polyvinyl alcohol. It was established the embedded cells continuously produced cephalosporins for 96 h with a yield similar to that of freely resting cells. The same strain gave significantly lower amounts of antibiotics when immobilized via the direct polymerization of acrylamide monomers [68].

The immobilization of the enzyme deacetoxycephalosporin and its analogs depends on diethylaminoethyl-ino ion-exchange chromatographic resin from L-α-aminoadipyl-L-cysteinyl-D-valine and its analogs. An incorporated enzyme reagent capable of continuously cyclizing, epimerizing, and expanding L-α-aminoadipyl-L-cysteinyl-D-valine and analogs was thereof derived from the prokaryotic β-lactam organisms *S. clavuligerus*, *S. cattleya*, and *S. lipmanii* [69].

*Streptomyces rimosus* cells were immobilized in Ca-Alg and used to produce oxytetracycline. The effects of the cultivation age, alginate content, and storage in CaCl_2_ were determined. According to the report of recurring periodic fermentations in shake bottles, a decent antibiotic yield was maintained for 28 days using 4% Ca-Alg. *Streptomyces rimosus* escape and its concentration inside the beads were affected by the Alg content and storage in a CaCl_2_ solution [70].

Penicillin acylase from *Streptomyces lavendulae* was embedded in epoxy-activated acrylic beads (*Eupergit C*). The sequential amendment of the matrix with bovine serum albumin leads to a novel biocatalytic system (ECPVA) with enhanced (1.5-fold) penicillin V hydrolysis activity compared to its soluble counterpart. This biocatalyst had a K m value of 7.6 mM, slightly higher than the K m for native acylase (3 mM) [71].

Enzymes from *Streptomyces clavuligerus* were immobilized on anion exchange resin to biosynthesize penicillins and cephalosporins. Jensen et al. evaluated this by plating 5 mL of salt-precipitated cell-free extract with a 1 mL volume. The production of penicillins and TDE buffer for the salt-precipitated cell-free extract was estimated to have a protein content of 0.065 g/mL of packed gel (0.081 g/g of wet dry gel). For all further studies, the gel was loaded up to 85%. However, only 50 mg/mL of the protein in the salt-precipitated cell-free extract did not bind to DEAE-trisacryl under these conditions [72].

## 5. Immobilization of Bacteria

In 2023, the global Immobilized Enzyme Reactor Market, according to reports estimated, reached USD 104 billion, and it is expected the grow to up to approximately USD 193 billion by 2031 [73]. Enzymatic production from benzylpenicillin (Pen G) 6-aminopenicillanic acid (6-APC), the enzyme penicillin G acylase (PGA) utilized, was immobilized on solid carriers, and the immobilized penicillin G acylase (IMPGA) was determined. Natural and semi-synthetic penicillins contain 6-aminopenicillanic acid. Diverse sorts of penicillin differ in the attached side chains [74].

The impregnation of PGA was performed via whole cells of E. coli in a chitosan-based carrier. First, cells were permeabilized with the surfactant N-cetyl-N,N,N-trimethylammonium bromide (CTAB) (0.1%, 45 min), which were immobilized with 50 mg/mL of the crosslinking agent GA, and 30 mg/mL of chitosan was used as a matrix. As a result, immobilization on chitosan decreased the penicillin G conversion by 13%, and the permeabilization of cells caused a 9% increase in penicillin G acylase conversion after a quarter of an hour compared to intact cells [75].

A method of immobilizing PGA includes the following steps: (1) Fe_3_O_4_ particles synthesized by a reversed microemulsion assay, followed by 3-(trimethoxysilyl) propyl methacrylate (KH-570) functionalization to improve the quality of dispersion and biocompatibility; (2) the magnetic composite scaffold was modified by grafting glycidyl methacrylate onto the surface; (3) the composite was utilized for covalent PGA attachment via an epoxy group. The optimal conditions were a glycidyl methacrylate content of 9%, leading to enzyme activity reaching 9208 U/g, an enzyme activity recovery rate equal to 88 mg/g, and an enzyme loading rate of 64%. The penicillin acylase obtained using this method has high enzymatic activity and good stability over time [76].

Penicillin G amidase/acylases microbial bases are an exceptional enzyme belonging to the structural superfamily of N-terminal nucleophilic hydrolases. The production of PGA from various microbial sources uses *E. coli* under improved environments. The minimum yield of wild strains was significantly enriched using various strain-improvement procedures, such as recombination and mutagenesis [77]. PGA from *Escherichia coli* was embedded in vinyl sulfone agarose. The final settings for immobilization were 1 M of sodium sulfate at pH 7, incubation at pH 10 for 3 h in glycerol and phenylacetic acid, and finishing via blocking with glycine or ethanolamine. This resulted in enzyme stability comparable to glyoxyl-PHC, with more than 55% of the originally proposed enzyme activity compared to 80% recovered using glyoxyl-PHC [78]. The covalent immobilization of penicillin G acylase from *Escherichia coli* was then performed. A total of 2.7 mg of pure biomolecule was included per gram of sepabusin using an enzymatic binding yield of 97%. Correspondingly, a supreme yield of 89% could be achieved by operating at a small enzyme load (0.14 g/kg). Then, free and embedded penicillin acylase trailed ordinary Michaelis–Menten kinetics, indicating the equivalent reaction mechanism in both cases [79]. The ultrastructure of hydrogels incorporated into scaffold cells, as well as the scattering of attachment of *E. coli* cells within polyacrylamide cryogels with different methods, were studied. The first immobilization sort had the greatest amount of biomass in the gel (protein) and a dramatic decline in cell viability. The second sort was unsuccessful in preserving the cells in the pores, and GDA treatment meaningfully diminished the viability index. The last method was the most sparing and preserved the population’s viability [80].

*Penicillin* acylases (penicillinamidohydrolases) are a group of biocatalysts that catalyze the scrupulous hydrolysis of the moderately firm side chain amide in penicillins and cephalosporins, parting the labile β-lactam disk intact. For several years, enzymes capable of this exceptional conversion (primarily penicillin acylase from *E. coli*) have been extensively investigated as industrial biocatalysts for modifying β-lactam antibiotics [81].

Penicillin acylase derived from *Bacillus megaterium* ATCC 14945 and marketable incremented enzyme from *Escherichia coli* for the synthesis of ampicillin and cephalexin allowing for DMSO, CH_3_OH, and sorbitol as organic co-solvents. The yield of ampicillin and cephalexin was higher for the enzyme from *B. megaterium*, 21.3% and 45.5%, correspondingly associated with 14.5 and 29.5% for the enzyme from *E. coli*. Sorbitol increased the yields of ampicillin and cephalexin by 28 and 20%, respectively, and the penicillin acylase activity was entirely improved in this reaction medium after 12 h. Oppositely, the yields were worse in 20% DMSO and 20% CH_3_OH, and the biomolecule underwent inactivation in these mediums [82].

The most advanced approach to date is the primary industrial strategy for converting cephalosporin C, involving a three-enzyme, three-pot process utilizing immobilized d-amino acid oxidase (DAAO), glutaryl-7-ACA acylase (GLA), and cephalosporin C deacetylase (CAH). Two parallel sequence reactions were ascribed: (1) cephalosporin C (CPC) was hydrolyzed to 7-ACA by CPCA, followed by the deacetylation of 7-ACA to D-7-ACA by CAH; (2) CPC was first hydrolyzed to deacetyl-CPC (D-CPC) by CAH and then de-α-aminoadipoylation of D-CPC to D-7-ACA by CPCA. Under an optimized biocatalyst process, a D-7-ACA yield of 78% was achieved within half an hour in a single reactor. The specific productivity of D-7-ACA reached 10.85 g/g/h/L, which is approximately four times higher than that of the traditional two-pot enzymatic process [83].

The covalent attachment of CPC acylase, utilizing LX-1000EP as the support, revealed the highest activity of 81 U/g, indicating its strong potential for industrial 7-ACA production. The optimal activity of the immobilized enzyme was observed at a pH range of 8.5 to 9.5 and increased with temperature up to 55 °C. Additionally, the immobilized CPC acylase exhibited decent stability within a pH range of 8 to 9.5 and at temperatures up to 40 °C, and a 7-ACA yield of 96.7% was obtained in 1 h [84].

Sklyarenko’s group reported the encapsulation of cephalosporin-acid synthetase (IECASA) from the recombinant *E. coli* strain to synthesize a high yield of ceftazidime (CEZ) relative to 7-amino-3-(5-methyl-l,3,4-thiadiazol-2-yl)thiomethyl-3-cephem-4-carboxylic acid (TDA), ranging from 92% to 95%. The final reaction mixture contained 65–85 mg/mL of CEZ, 4–5 mg/mL of unreacted TDA, and 40–60 mg/mL of by-product. After 25 sequences, the residual activity of IECASA was approximately 83  ±  2% of its initial value. The half-inactivation period of IECASA was forecasted to be 85 series of CEZ synthesis [85,86].

Wei et al. suggested the covalent attachment of CCA on an epoxy-activated support, LX1000-EPC4 (EP), or its derivatives, EP-polyethyleneimine (EP-PEI) and EP-ethylenediamine (EP-EDA), which possessed a cationic surface. The enzyme immobilized on EP-PEI demonstrated significantly better thermal stability, with a stabilization factor 32 times greater than the native enzyme, compared to a stabilization factor 5.5 times greater for EP-EDA and 1.7 times greater for EP. The adsorption of CCA on the EP-PEI support was strong and reversible, with the enzyme being fully desorbed using a high concentration of NaCl (2 M) at pH 3. The EP-PEI support could be recycled and reused for further enzyme immobilization [87]. The rapid catalytic velocity during the initial conversion stage led to a sharp intraparticle pH gradient, which was likely the primary cause of low operational stability. To address this, a novel two-stage catalytic process was recently reported. Reducing the reaction speed in stage I at a low temperature, fermentative activity was better preserved, while a higher reaction temperature during stage II shortened the overall catalysis duration. This two-stage process (with a temperature shift from 10 °C to 37 °C after half an hour) significantly improved stability, enabling 29 batches compared to just 5 in the single-temperature process at 37 °C, and also reduced the catalytic period compared to the reaction at 10 °C (40 vs. 70 min) [88].

The acylase from *Arthrobacter viscosus* was immobilized and investigated to determine its effectiveness in the enzymatic synthesis of various cephalosporins. Its performance was then compared with that of penicillin G acylase. Biocatalysts immobilized on hydrophilic resins, such as glyoxyl-agarose (activated with aldehyde groups), exhibited superior synthetic performance compared to those immobilized on hydrophobic acrylic epoxy supports, like Eupergit C. The acylase from *Arthrobacter viscosus* demonstrated better synthetic performance than the penicillin G acylase from *E. coli* [89].

It is well known that a PVA-GA acidic medium is needed to synthesize hydrogel. Thus, *Bacillus pseudomycoides* were immobilized in a PVA/GA gel. Studies showed that a GA/PVA mass ratio of 0.03 to 1 and pH 2 are necessary for hydrogel synthesis. Overall, the results of this laboratory work show that 32 and 86% BOD reduction was observed in GA/PVA hydrogel and BI GA/PVA gel, respectively, and free bacterial cells were metabolically active, resulting in BOD reduction by 87% [90]. The bacterial immobilization method uses crosslinking of PVA with sodium sulfate. Residue occurs after each addition of nutrients, and the pH in the reactor is at a low level (<3.5). From the fifth day to the eight day, the pH is adjusted using a NaHCO_3_ solution. As a result, from day sixteen onwards, the reactor performance increases and stabilizes at 80–87% [91].

The immobilization of microorganisms on hydrogels (CPVA) and its foam (CPVA) involves *Bacillus licheniformis*, *Rhodococcus erythropolishas*, and *Pseudomonas xanthomarina* microorganisms. The crosslinked CPVA carrier showed thermostability and improved microbial performance in hydrocarbon deterioration compared to the CPVAF carrier. SEM analysis revealed the existence of extracellular structures that may play an important role in the stability of cell immobilization on gels. It was established that the prospective usage of CPVA and PVAF individually as cell carriers and Bios independently could help to release cells [92]. Boric acid and sodium sulfate were used to make PVA-Ca gel beads to immobilize Anammox bacteria. Two different continuously stirred reactors (CSTR) were used to make gel beads. In reactor 1, only inoculum sludge with Anammox biomass was used, and reactor 2 was used as a reference. As a result, after 20 days, the Anammox procedure was noticed in reactor 1, while reactor 2 showed that aerobic oxidizing bacteria (AOB) and nitrite oxidizing bacteria (NOB) prevailed in PVA-Ca gel beads [93].

Alg crosslinked with Ca^2+^ or other salts is not stable throughout cultivation due to diffusion out of ions and swelling and, as a result, loose mechanical properties and the following loss of cells. The combination of Alg and PVA provides additional hydrogen bonding between polymers and reinforces the stability of a carrier. Thus, Winkler’s group encapsulated ammonia-oxidizing archaea bacteria in tiny (~2.5 mm) and larger (~4.7 mm) PVA-sodium alginate hydrogel beads linked with various agents (calcium, barium, light, or sulfate). Consequently, PVA(6%)-Alg sulfate-bound pellets were the most stable, with diffusion coefficients 2–3 times higher in hydrogels than in pellets. Despite the extended lag phase in tiny beads, the introduced ammonia-oxidizing archaea maintained a high volumetric rate of ammonia oxidation [94].

## 6. Production of Antibiotics

Antibiotics of bacterial origin can be divided into several groups: tyrotricin, gramicidins, polymyxins, bacitracins, nisin, etc. The antibiotics tyrotricin and gramicidin C (S) were obtained from *Bacillus brevis*. The conditions for forming gramicidin C were as follows: gramicidin C was preserved for a long time on media containing meat hydrolysate or on 10% yeast hydrolysate under surface cultivation of the organism and a temperature of about 40 °C. Under the aforementioned conditions, up to 2000 µg/mL of gramicidin C is formed [95]. We summarize data related to various antibiotics’ formation via biotechnological usage of the bacteria genus *Bacillus* in Table 2. This study was devoted to estimating the antimicrobial activity of *Lactococcus Lactis (L. Lactis)* against *Staphylococcus aureus (S. aureus)*. The study of their antibiotic sensitivity was tested on Man rogosa Sharp (MRS) agar, which is more commonly adapted for the study of the antibacterial activity of *L. lactis.* The strain *L. Lactis* ssp. cremoris showed highly potent antibacterial activity, reaching 40 ± 3 mM against *S. aureus*, while the other strains (*L.lactis subsp*, *Lactis diacetylactis*, *L. lactis* subsp. *lactis)* showed slightly moderate antioxidant activity ((10.56 ± 3 mM) 1.28%–26.29 ± 0.05%). Consequently, these strains of *L. Lactis* were resilient to cefotaxime and streptomycin and sensitive to ampicillin, penicillin G, vancomycin, gentamicin 500, tetracycline, and chloramphenicol [96].

The deep cultivation of *Bacillus brevis* producer on synthetic media to produce gramicidin C in a 10-L fermenter was conducted for 42 h under oxygen-limited growth conditions. As a result, a culture containing 38 mg/mL of biomass and 5.28 mg/mL of gramicidin C was achieved with impurities of 8.44% of gramicidin C [97].

Shmygarev et al. described a method for producing the antibiotic amikumacin A that involves culturing a *Bacillus pumilus* strain producing Ami (amikumacin A inhibitor), which is cultured at 28 °C in SYC medium and cultivated in 100 mL of medium thermostatically with a rotation speed of 250 rpm. The cells were centrifuged at 10 K× *g* for 600 s. Thus, an inactivated analog of Ami 5 was generated—the modified Cbz-LeuCG-Ami 5 derivative did not hinder cell growth up to an accumulative concentration of 50 mg/L [98].

The antibacterial peptide subtilosin A is one of the nine bacteriocins described in B. subtilis isolated from the plant material *Passiflora edulis*. The strain was cultured on Petri dishes with Nutrient Agar M001, GRM agar, or starch agar at 30 °C and 36 °C for 24 h. The cultures were screened using the McFarland bacterial turbidity standard, and the activity reached 102,400 AU/mL. Subtilosin A. The isolated peptide (3.4 kDa) was active against *Bacillus anthracis*, *Bacillus cereus*, *Staphylococcus aureus*, and *Listeria monocytogenes*, which makes it attractive for use as an available naturally occurring antimicrobial [99].

Currently, subtilosin can be produced using an animal MRS medium as a carbon source with two animal peptones. An intensification in cell number, with an increase in subtilosin activity, occurred when cells were cultured in AF-MRS supplemented with 0.4% K_2_HPO_4_ and 0.02% MgSO_4_. Subtilosin production increased from 30 to 320 U/mL when stirred on an orbital shaker at 300 rpm [100].

Subtilisins (EC3.4.21.62) are proteases secreted by *Bacillus subtilis* (MTCC 441). Optimal growth conditions for *B. subtilis* MTCC 441 were found at 37 °C and pH 7.5. The best medium configuration for subtilisin production was as follows: 0.675% yeast extract, 0.44% peptone, 0.6% NaCl, 1.075% casein, and 0.5% glucose. The foreseen and actual responses were 181 U/mg (at a desirability of 0.87) and 185.7 U/mg, respectively [101]. The dipeptide antimicrobial active ingredient bacilysin revealed efficiency against a wide range of bacteria [102]. Bacilysin containing active epoxy and keto functionalities, an antibiotic produced by *Bacillus* species, is active against various microorganisms and the *Candida albicans* fungi due to its simple structure and excellent mechanism of action [103]. Table 3 contains comprehensive data attributed to microorganisms that are sensitive to natural antibiotics.

Nguyen et al. studied the antibiotic resistance and biofilm development of *Escherichia coli* in a Vietnamese fish-processing plant. Antibiotic sensitivity illustrated the maximum resistance to sulfamethoxazole/trimethoprim (45%), followed by tetracycline (39%). These isolates of E. coli were responsive to meropenem and fosfomycin and marginally aqueous acidic and hypochlorous at 40 mg/L, providing active chlorine or sodium hypochlorite with 0.1 g/L of available chlorine, resulting in a substantial decrease in biofilm mass [104].

## 7. Immobilised Bacterial Cells

Fareed et al. immobilized microbial cells to offer a favorable potential strategy in almost all processes. In addition, immobilized Rhizobacterium cells are better able to endure poisonous chemicals, solvents, heavy metals, and high temperatures and pH. Bacterial cells were cubed in 10 g of agar dissolved in 1 L of 0.9% NaCl solution. For instance, in specimens inoculated with cells immobilized in agar at 30 °C and at 4 °C, the deterioration of immobilized cells was 80–90%. On the other side, the suspension of cells metabolized up to 60% of carbamates at 4 °C, but degradation up to 80% was recorded for a cell suspension at 30 °C. The immobilized cells of a novel microorganism amplified the degradation of N-methylated carbamates under low-temperature conditions [105].

The investigation of suspensions of *Pseudomonas guariconensis* and bacterial cells immobilized within calcium alginate gels revealed the following activity: the immobilized cells were capable of degrading the dye with an efficiency of 91% compared with 86% for cell suspensions. The incorporation of bacterial cells was displayed to advance the enzymatic activity and permanency of the bacterial cells, explaining the variances in bacterial strain dye degradation productivity. In addition, *Bacillus* sp. JF4 was embedded into pellets of PVA and Alg-Ca, and activated carbon was investigated to determine the decolorization of reactive blue 19 (RB 19). Incorporating the bacterial strain achieved a degradation efficiency of 100% compared to 92.1% for the bacterial suspension and illustrated good tolerance to large concentrations of RB 19 dye [106].

Börner et al., for the first time, utilized activated aldehyde groups of polyethyleneimine (PEI-al) and PVA-al as crosslinking agents to immobilize *Clostridium acetobutylicum* DSM 792. The obtained cryogels preserved cell viability and demonstrated cryogel glucose consumption and solvent production in a liquid medium. Solvent production was 2.7-fold higher in immobilized cells compared to free cells, and the cryogel could be used 3–5 times with partially or completely fresh medium, achieving a maximum butanol concentration in the medium of 18.2 g/L and a yield of 0.41 (g/g) per cycle [107].

Partovinia and Vatankhah reported on a combination of Alg-PVA gels used to immobilize cells. Sodium alginate (20 g/L) and PVA (125 g/L) were prepared in distilled water at conventional temperature and 80 °C, respectively. Thus, the degree of released cells from the microbeads was measured gravimetrically, and the quantity was nearly 2 mg/L, which was equal to 4% of the original incorporated cells in the alginate/PVA beads. The microbial cells were successfully entrapped by the alginate/PVA hybrid matrix [108]. However, the applied method is inaccurate, as polyelectrolyte hydrogels tend to swell or collapse in certain conditions. Viable cells release degradation products, which change the microenvironment and may trigger gel shrinkage. Therefore, to evaluate cell content in a hydrogel, it is better to use spectrophotometric monitoring, which provides more accurate quantitative data.

Next, this crosslinking approach via PEI-al and PVA-al was applied to cryogel preparation. The viability of cells after covalent crosslinking was confirmed using an MTT assay, as life–dead assays are not practical for the quantitative evaluation of cells. However, this method is widely applied to illustrate the overall picture of immobilized cells [109].

*Bacillus subtilis* cells were immobilized in PVA–cryogel beads by applying the well-known cycling freeze–thaw method. The conditions were optimized to ensure both high thermal and mechanical stability. Bacterial spores, Na-Alg, and bacterial cellulose accelerated the solidification of the gels and changed their porosity [110].

This study crosslinked lactic acid bacteria using GA, oxidized dextran, and activated polyethylenimine/modified PEI/PVA and used the monolith as a continuous and side reactor, where 19.7 g/L 3-hydroxypropionic acid was obtained at a speed of 9.1 g/L/h, and the yield equalled 77 mol% [111].

*E. coli* cell-immobilized PVA hydrogel membranes (ECI-PVAHM) were prepared using a 10% PVA solution. The bacteria-loaded film exhibited high water resistance, the tensile strength was 0.66–0.90 MPa, elongation was 300–390%, and swelling was 330–800%. The efficient bacterial content of ECI-PVAHM was 2.375 × 10^9^ up to 10^10^ CFU/g recalculated relatively dry cell weight, which did not affect the original crystal structure of PVAHM. The biofilm possessed a pore size arrangement of 0.2–1.0 μm, the cells were uninterruptedly cultured for nearly 3 weeks, and the medium was reintroduced two times a day. The growth efficiency was about 91% after 40 cycles [112].

Immobilized enzymes may have application in water treatment for fine bacteria removal. The immobilization of lysozyme and immobilization systems and the efficiency of bacterial removal from poly(HEMA-EH) cryogels are described. The maximum loading of lysozyme was 43.56 mg/g of cryogel. *Micrococcus lysodeikticus* bacteria were used to determine the bactericidal effect of the immobilized lysozyme [113].

Zaushitsyna and colleagues investigated the storage stability of living cells crosslinked within a cryogel structure. The activity of cryoPEI-al and PVA-al decreased insignificantly, retaining almost 80% of the initial enzyme activity after 30 days. Cells crosslinked with the lowest (0.45%) and highest (1.3%) amount of polymer exhibited almost equal enzyme activity. It was established that synthetic polymers are mild on the cells, and the amount of polymer does not affect metabolic activity [114].

Glucose isomerases in *E. coli* are immobilized on the modified diatomite surface as a carrier, and 74.1% of suspension cell activity to enhance the operational stability was recovered after immobilization. Then, the immobilized cells reserved 86.2% of the original transformation activity within 40 series, and the yield of D-fructose was more than 42% at 60 °C [115].

**Figure 1:**
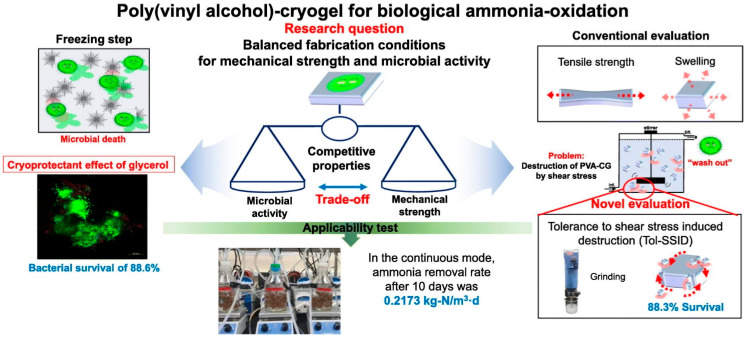
The experimental set-up of PVA–cryogels with immobilized nitrifier use and the effect of various mixing regimes on cell survival. Reproduced with permission [116].

*Paenibacillus phytohabitans* KG was immobilized on PVA cryogels as follows: Song et al. studied poly (vinyl alcohol)–cryogels (PVA-CG), which had improved parameters. The mechanical strength of freezing–thawing cycles (FTCs) was remarkable, featuring high friction, and the PVA concentration in PVA-HG was 10%. The tolerance to SSID (Tol-SSID) was 88.3% at two FTCs. The tensile strength of porous material was 0.59 kPa; 50 g/L of glycerol was added to maintain respiratory activity, the activity of immobilized nitrifying bacteria was 0.097 mg-oxygen/g-OCC min, and the substantial survival rate was 88.6% (Figure 1) [116].

*Ganoderma lucidum* adsorbed on polyurethane foam showed enhanced performance in the uninterrupted process of exopolysaccharide EPS in repeated batch fermentation. A total of seven groups were fermented sequentially in shake flasks at an 80% broth ratio for 55 days and on the 13th day. The throughput in the immobilized culture was 0.045 g/L on the first day and was larger compared to the free-floating culture (0.029 g/L on day 1) [117].

## 8. Immobilization of Fungal Cells

**Figure 2:**
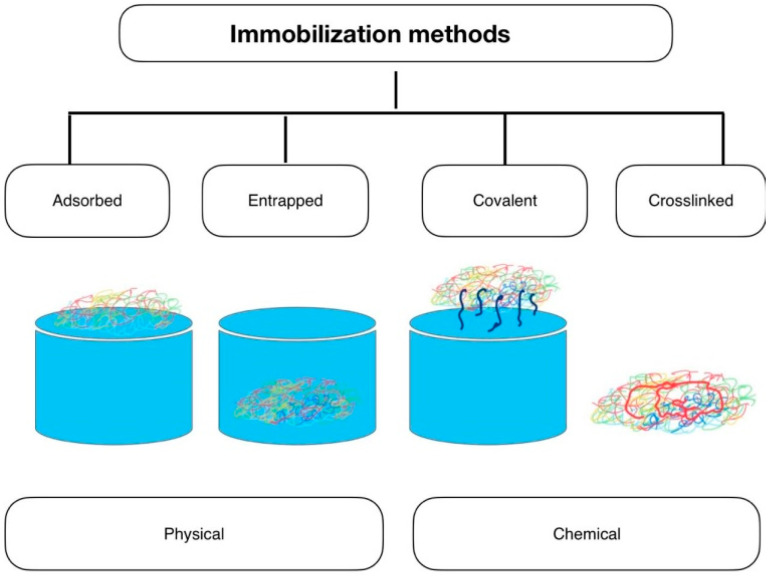
A general schema of the classification of microorganism-immobilization methods.

Fungal cells can be adsorbed on the surface of solid inorganic or organic/polymeric carrier via covalent attachment or via physical adsorption hydrogen bonding, electrostatic and hydrophobic interactions. Cells can be entrapped within the covalently or physically cross-linked hydrogel structure which is illustrated in Figure 2. Cells may be cross-linked using bifunctional derivatives such as PEG-diglycidyl ether, glutaraldehyde, bismaleimide Crosslinkers etc.

The survival rates of immobilized and released spores when making traps by suspending spores in a polymer and freezing them at 18 °C for 24 h were the same, indicating that there was no poisonous effect of the polymer and acquaintance to low temperatures did not destroy the spore structure; spore leakage after 24 h was different in different cases of immobilization—for example, the lowest was 15%, and the highest was 12%. After 96 h, the lowest degree of spore immobilization was 10%, then 15%, and the highest was 12%, which persisted up to 120 h [118]. Poly(2-hydroxyethyl methacrylate) [Pc-PHEMA] cryogel disks were prepared using the filamentous fungus *Penicillium chrysogenum* as Pc-PHEMA biosorbent, and 200 hybrid cryogels had a biosorption efficiency of more than 82.8% for Cd^2+^, Pb^2+^, and Zn^2+^ at heavy metal concentrations in the range from 0.1 to 2.5 mg/L [119].

Allyl glycidyl ether monomer with acrylamide was used for covalent encapsulation via cryopolymerization and immobilized α-amylase from *Aspergillus oryzae*. Accordingly, maximum starch hydrolysis was accomplished at pH 5, immobilized enzyme content of 111 mg of amylase/cryogel, a starch solution concentration of 45 g/L, and a temperature of 35 °C. In this case, the immobilized enzyme showed a degree of conversion of 68–97%, depending on the pH and temperature [120].

Biocatalysts are immobilized on *Rhizopus oryzae* cells, which are encapsulated in PVA cryogel to convert L(+)-lactic acid (LA). Under batch-process conditions, the immobilized biocatalyst produced LA from glucose and starch hydrolysate with yields of 94% and 78%, respectively. Under semi-stationary conditions, yields of 52% and 45% were gained from the corresponding substrates. The utmost productivity of the process (up to 173 g/L) was achieved under semi-stationary conditions. Starch (5–70 g/L) was converted to lactic acid via physical embedding on an *R. oryzae* carrier. The possibility of long-term operation (480 h) of immobilized biocatalysts was demonstrated [121].

Fungi and bacteria were used as cells for immobilization on PVA cryogels. After 200 h, the amount of accumulated LA was the same for these fungal (920 g) and bacterial (895 g) biocatalysts. The rate of conversion of the substrate into the product of the fungal biocatalyst was two times higher (0.92 g of LA per g of glucose) compared to that of the bacterial one. The half-life of the incorporated fungal biocatalyst was 80 days (96 operating cycles), which is 10 times greater than the half-life of the bacterial enzyme [122].

## 9. Advances in Enzyme Immobilization

Enzymes play crucial roles across a spectrum of industrial divisions and are integral modules of numerous industrial high-added-value products. Incorporated enzymes exhibit heightened resilience to environmental fluctuations and may be conveniently regenerated and reprocessed compared to their nonimmobilized counterparts. The major advantage of immobilization lies in shielding the biocatalyst from adverse environmental circumstances, such as high temperatures and extreme pH levels. Immobilized enzymes have applications in various large-scale industries, including but not limited to medical, food, detergent, textile, pharmaceutical, and water treatment plants [123].

This review offers an overview of (multi)enzyme immobilization, encompassing a thorough assessment of cell inclusion into hydrogel structure techniques and carriers, biocatalyst metrics, the influence of crucial carrier features on biocatalyst efficiency, trends in diminishment, and expressive examples showcasing biocatalytic applications [124].

While conventional immobilization methods, such as intramolecular chemical linking, sorption, encapsulation, and entrapment, are abundant in the literature, the current body of research reports falls short in addressing state-of-the-art smart chemistry approaches to immobilization. The work by Liu et al. fills this gap by focusing on the emerging field of surface functional entities that interact among carrier interface and the given enzyme, thereby providing a more comprehensive understanding of modern immobilization strategies [125].

Laccase enzymes belong to oxidoreductases and are one of the valuable enzymes that catalyze a range of phenolic compounds. Cryogel based on 2-Hydroxyethyl methacrylate co polymer with 1-vinylimidazole as an affinity ligand was ascribed for the biosynthesis of laccase from *Aspergillus niger*. The purification coefficient under optimal conditions was 10.53, and the recovery of the enzyme from the fermentation medium was 86.7% [126].

Trametes pubescens laccase was immobilized on wide-pore PVA cryogels. The enzymes were obtained and purified. The resulting laccase (yield 40%) showed an efficiency of 46.4 units/mg and protein content equal to 12.5 g/L. After, the biomolecule was covalently attached to functionalized cryogel beads. The time dependency of the adsorption process and the loading of the enzyme with the carrier material were established (5.2 g/kg of macroporous hydrogel) [127].

Recombinant laccase POXA1b from *Pleurotus ostreatus* was immobilized on epoxy-activated poly(meth)acrylate beads and adjusted using the response surface method. The immobilized laccase was approbated in a fruit juice clarification process, achieving a phenol reduction of up to 45% without compromising flavanone content [128].

**Figure 3:**
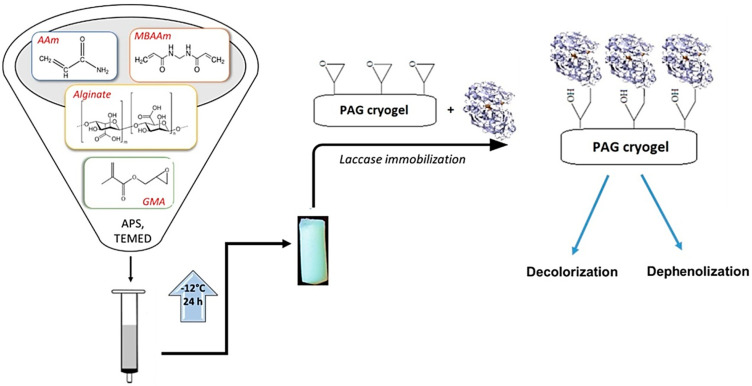
The scheme of covalent laccase immobilization on polyacrylamide-alginate cryogel. Adapted with permission [129].

Trametes versicolor laccase enzyme was immobilized on glycidyl methacrylate-functionalized polyacrylamide-arginate (pAAm-Arg) cryogels, and maximum loading capacity (68.7 ± 1.45 g/kg) was observed at pH 3 and 25 °C (Figure 3) [129].

Hybrid cryogel columns are coated with PEI and poly(HEMA-co-GMA), and the optimal activity of native and immobilized enzymes is pH 6. The optimal temperature for the free enzyme was 55 °C, while for immobilized PHG/PI-Xyl, it was 60 °C. PHG/PI-Xyl showed outstanding thermal stability, with an initial activity of 53% at 60 °C for 180 min and 32% in the absence of polymer Xyl. /PI-Xyl retained 49% and 69% of the initial activity of free and immobilized xylanase after a month of storage at conventional temperature, while PHG/PI-Xyl reserved about 58% of the initial activity after 10 repeated series, Km of 4.05 mg/mL for free xylan and PHG/PI-Xyl of 2.6 mg/mL and Vmax of 133 U/mL and 189 U/mL, respectively [130].

Cryogel composites with diallyldimethylammonium chloride as a crosslinker were embedded in a colored metal–organic framework (MOF) to rapidly form a polydopamine membrane. Then, immobilized trypsin showed an effective immobilization time of 1 h, a wide range of catalytic temperatures of 25–65 °C and pH 6–10, and shelf stability (one month), illustrating more than 80% lasting activity. As a result, the Km and Vmax values were 18 g/L and 17 μg/mL/min, respectively, and 14 g/L and 16 μg/mL/min for the native trypsin solution, respectively [131].

**Figure 4:**
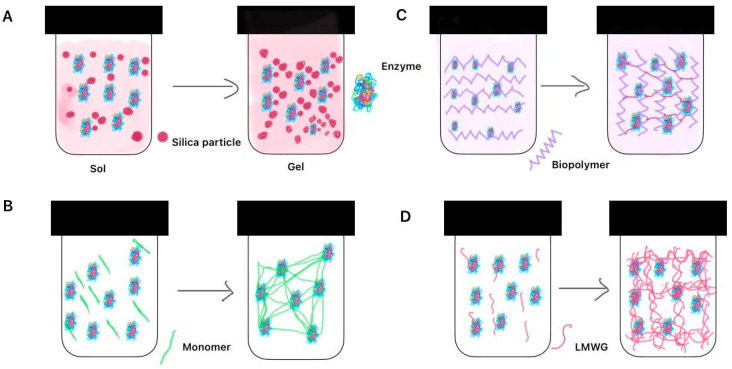
A schematic illustration of enzyme entrapment in gels. (**A**) Sol–gel (silica gel); (**B**) polymerization; (**C**) crosslinking biopolymer; (**D**) supra-molecular assembly (low-molecular-weight gel).

An illustration of various strategies of enzyme immobilization: on a surface of SiO_2_ or other types of inorganic particles; cross-linked premade synthetic polymers and natural polymers in gels; entrapment to the hydrogel structure as a result of self-assembly of low molecular weight organic molecules (Figure 4).

When immobilizing catalase, cryogels (poly(HEMA-GMA)) with different amounts of GMA were used. After 9 h, the adsorption capacity of catalase when using cryogel was 299 ± 9.9 mg/g [132].

L-asparaginase was immobilized on poly(HEMA-GMA) cryogel with a yield of 68.8% and activity recovery of 69.3%, and the storage steadiness and regeneration of the immobilized enzymes were roughly 54% and 52% of their native activity after 28 days at room temperature and 10 sequences, respectively [133].

**Figure 5:**
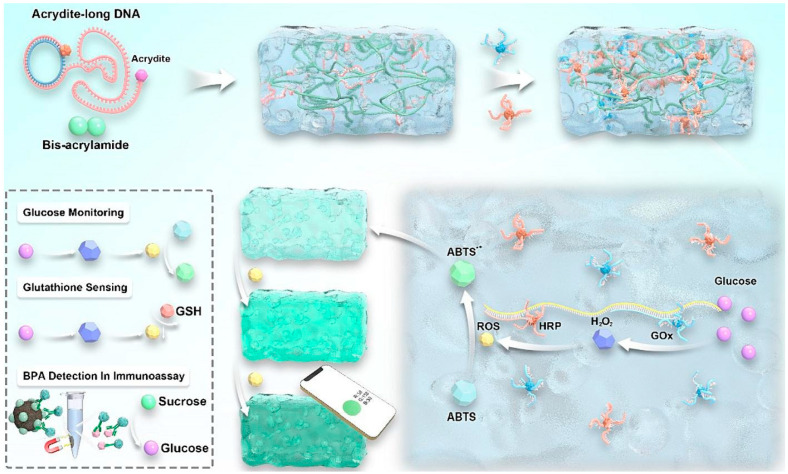
A scheme of gel preparation and following enzyme immobilization to fabricate an efficient platform with enhanced catalytic activity for portable glucose biosensing. Adapted with permission [133].

Chinese researchers proposed a novel but quite expensive approach to address the challenges of enzyme immobilization, such as enzyme connection to the surface and reduced catalytic performance, in DNA cryogel bioanalysis. This was achieved via non-covalent but strong linking between DNA and DNA, leading to the optimized microporous structure of the cryogel. Utilizing this method, cascade enzyme-based cryogel sensors were developed for glucose concentration evaluation and glutathione detection, demonstrating exceptional sensitivity and accuracy. Furthermore, by integrating with a modern phone, a recognizing platform was created to quickly estimate various target molecules in different mediums, highlighting its significant prospective for onsite monitoring and early disease identification (Figure 5) [133].

The enzyme L-asparaginase was covalently attached to magnetic nanoparticles utilizing APTES, providing amino functionality to the surface of nanoparticles. These nanoparticles have enhanced binding affinity to the enzyme L-asparaginase, which in turn led to the increased thermal stability of the enzyme by up to four times at 70 °C compared to the native solution of the enzyme. Moreover, it can be reused for up to five cycles. Kinetic parameters showed an increase in V max, Km, and catalytic effectivity by ~38% compared to the biomolecule in solution [134].

Covalently binding l-asparaginase in biodegradable nanoparticles (NPs) might be an encouraging approach to address the aforementioned problems related to this enzyme drug. Chitosan nanoparticles can be used as an effective delivery vehicle for L-asparaginase, and the thermal and pH permanence of l-asparaginase were also enhanced upon immobilization. The biomolecule is stable at pH 7.5–10 and a temperature of 60 °C. The capture productivity and charging aptitude of the synthesized nanoparticles were 72% and 53%, respectively [135].

It is worth considering other cases related to L-asparaginase immobilization. Nanoparticles of amino-(Fe_3_O_4_/SiO_2_/NH_2_) and carboxyl-functionalized (Fe_3_O_4_/SiO_2_/COOH) were used to immobilize L-asparaginase. The chemically attached L-asparaginase demonstrated superior stability within the process for 3 h at 50 °C. Thus, during the storage, native L-asparaginase reserved only 30% of its original activity for 4 weeks at 4 °C, while Fe_3_O_4_/SiO_2_/NH_2_@L-asparaginase and Fe_3_O_4_/SiO_2_/COOH@ L-asparaginase preserved more than 78.9% and 56.5% of the starting activities under identical experimental settings, respectively. Moreover, Fe_3_O_4_/SiO_2_/NH_2_@L-asparaginase (77.2%) and Fe_3_O_4_/SiO_2_/COOH@L-asparaginase (57.4%) had outstanding working stability after 17 repeated sequences [136].

Magnetite nanoparticles coated with L-Lysine (L-LYS@Fe_3_O_4_) were used to incorporate recombinant *E. coli* fabricating extracellular L-asparaginase II. The used L-lys@Fe_3_O_4_ nanoparticles had a size of ~12.5–22 nm and saturation magnetization of 42 eme/g. The immobilized cells were magnetically sensitive and completely assembled upon applying an external magnetic field (the immobilization efficiency was 100%). The magnetic immobilization resulted in a slight (4.3%) decrease in the plasmid-containing cell community, but this value was not statistically significant [137].

Programmed DNA interactions have been developed as a promising solution for enzyme immobilization to significantly reduce leakage. In addition, the porous structure of the hydrogel, by freezing, significantly improves its sensitivity and mechanical properties. As a proof of concept, DNA cryogels with adjustable spacing between cascade enzymes were developed to increase the local concentration of intermediates and reduce energy barriers to mass transfer for direct glucose monitoring and glutathione measurement [138].

**Figure 6:**
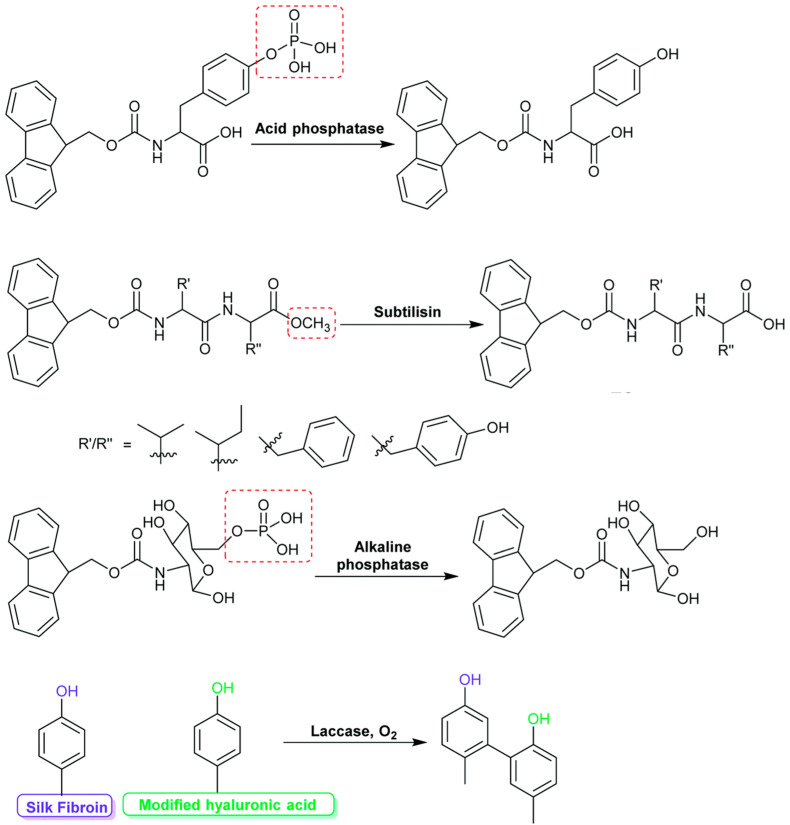
Examples of chemical reaction schemes for laccase enzyme-assisted gel formation. Reproduced with permission [139].

Another method to support the mild and efficient incorporation of enzymes into a hydrogel structure is the self-assembly of peptide derivatives. Motivated by the self-assembly processes in living cells, exertions have emerged to restrain biocatalysts on flat or decorated surfaces to achieve spatial regulation over molecular gelator generation and supramolecular nanostructure self-assembly. This report demonstrates the use of enzymes immobilized on magnetic Fe_3_O_4_ nanoparticles with PEG-COOH surface groups to restrict the beginning of peptide self-assembly into nanofibers, stabilizing the NPs. This method applies to two systems: an equilibrium biocatalytic arrangement, which forms firm gels, and a nonequilibrium system with a fixed period. The description of the gels reveals that self-assembly happens at the enzymes’ (Chymotrypsin from bovine and thermolysin from Bacillus thermoproteolyticus) immobilization sites on the NPs, resulting in hydrogels having a “hub-and-spoke” morphology (Figure 6) where nanofibers are connected via enzyme–NP conjugates. This NP-controlled organization of self-assembled nanofibers significantly enhances the shear strength of hydrogel systems and greatly extends the stability of hydrogels in the nonequilibrium system [139].

Acid phosphatase was used to hydrolyze phosphate-functionalized molecules. The enzyme was trapped inside the self-organizing nanofibers in the hydrogel. It retained 75% of its activity, while the embedded phosphatase revealed a remaining activity of 35% compared with the native aqueous enzyme solution. However, the immobilized version reserved 80% activity after 100 batch cycles at 30 °C. Moreover, the immobilized phosphatase engaged a primary activity of 60% conversion for 15–20 h in an uninterrupted process at 60 °C. The hydrolytic efficiency of the subtilisin enzyme was utilized to convert Fmoc dipeptides into starting acids and initiate the formation of highly ordered gel structures [140].

Effective nanobiocatalysts were developed using the laccase chemically attached to polymer/graphene hydrogel supports. These composite hydrogels were created through a novel, simple, and versatile method by self-assembling reduced graphene oxide platelets within a polymer latex matrix. The reduction in graphene oxide acted as the dynamic force for this self-assembly phenomenon. The adaptability of this procedure was demonstrated by incorporating various copolymers, which allowed variations in the hydrogels’ mechanical properties and functionalities (OH and Br groups). These variations led to different relations with the reduced graphene oxide platelets and, subsequently, different hydrogel morphologies [141].

The Fmoc-PhePhe hydrogel served several primary purposes: first, as a matrix embedding the enzyme HRP during the self-assembly of Fmoc-PhePhe peptides; second, as a durable substance for cell adhesion. It was reported that HRP was stably immobilized within the peptide hydrogel and maintained its inherent bioactivity toward hydrogen peroxide [142].

A DNA–protein hybrid scaffold was engineered using a programmable assembly method, serving as a biomimetic physiological environment for effective enzyme encapsulation. A double-stranded DNA building block, precisely modified with biotin functionality, was placed in contact with streptavidin, which triggered the development of the DNA–protein hybrid gel. This biocompatible matrix possessed a flower-like porous morphology with an average size of 6.7 ± 2.1 μm. It acted as a reservoir structure for enzyme encapsulation alcohol oxidase, retaining 78% of its activity after 24 h. The encapsulated enzyme demonstrated enhanced stability in various denaturing conditions, maintaining 60% activity after 30 min at 55 °C. Additionally, the enzyme preserved its full effectiveness after five freeze–thaw cycles and remained active despite being suspended in solutions containing organic solvents [143].

**Figure 7:**
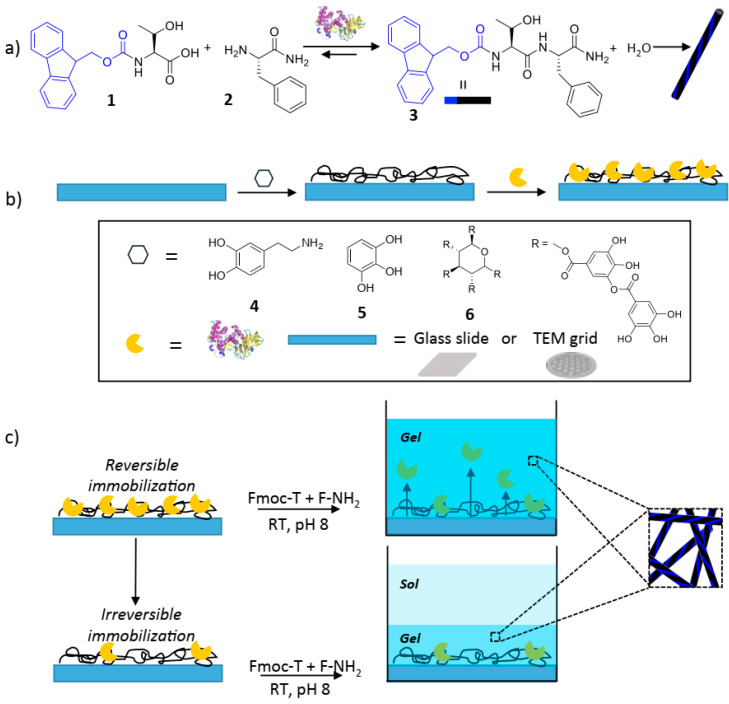
The process of catalyzing the conversion of pre-gelators and the surface modification for enzyme immobilization: (**a**) the transformation of the pre-gelators Fmoc-serin (1) and PheC(=O) NH_2_ (2) into the gelator Fmoc-Ser-Phe-C(=O)NH_2_ (3) catalyzed by thermolysin. (**b**) Interface alteration involves using (4) polydopamine, (5) **pyrogallol**, or (6) polyphenols followed by enzyme immobilization. (**c**) Reversible and irreversible biomolecule immobilization on improved surfaces is depicted for bio-catalytic self-assembly. Adapted with permission [144].

The biocatalytic regulation of molecular self-assembly offers a powerful method for creating advanced biomaterials, facilitating flexible enzyme-mediated adjustments to material structure and properties and supporting various biomedical applications. Authors of recent research modified surfaces with bioinspired polydopamine and polyphenol coatings to investigate how enzyme localization on the surface and enzyme release from the surface influence the self-assembly process. The structures of dopamine (4) (Figure 7), pyrogallol (5), and tannic acid (6) are shown in the inset, along with the thermolysin formula. The quantity of galloyl groups per tannic acid might range from 2 to 12, depending on the plant source. Penta-m-digalloyl-glucose from oak gall nuts is depicted (Figure 7b). Enzymes that are temporality bound to the surface catalyze the coupling of the pre-gelators Fmoc-Ser (1) and PheC(=O)-NH_2_ (2), leading to the materialization of a bulk gel. Enzymes permanently bound to the surface maintain their efficiency after washing and can only catalyze the transformation of the pre-gelators at the surface, facilitating confined self-assembly of the gelator Fmoc-Ser-PheC(=O)-NH_2_ into nanofibers (Figure 7c) [144].

Conte et al. utilized innovative polyphenol surface coatings to immobilize thermolysin, demonstrating that their effectiveness is on par with polydopamine coatings. They provide evidence that surface-mediated bulk gelation results from enzymes released into a pre-gelator solution rather than biomolecules attached to the surface, as suggested before for a similar arrangement. Researchers have proposed that biocatalysts may be reversibly or irreversibly attached on a surface, with the amounts of these inhabitants controllable through surface treatment. While polypeptide desorption (i.e., reversible adsorption) is commonly observed on various polymers, metals, and metal oxides, there is no general rule ensuring that the released enzymes maintain their conformation and activity. Conversely, common surface-functionalization techniques using silanes, thiols, or bifunctional linkers are considered to irreversibly covalently bond polypeptides, preventing significant protein desorption or leakage [145].

When polyphenol/polydopamine-coated models undertake a slight washing process, as shown in Figure 7, reversibly adsorbed thermolysin lasting on the surface illustrated gradual desorption to trigger catalyzed gel formation of Fmoc-SerPheC(=O)-NH_2_ in the solution, facilitating bulk gel formation. However, more extensive washing ensures that loosely bound enzymes are removed, leaving only irreversibly bound enzymes. These enzymes can still catalyze the conversion of pre-gelators, but only near the surface. The immobilization procedure resulted in approximately half a monolayer of active enzymes, enabling localized self-assembly of nanofibers without bulk gelation [145].

The disadvantage of using some self-assembled gels lies in enzyme immobilization in long-term storage. Silica nanoparticles (SiNPs) modified chemically with alkaline phosphatase (SiNPs@AP) promote the localized development of self-assembly of peptide nanofibers by dephosphorylating Fmoc–PhePhe-p-Tyr peptides (Fmoc: fluorenylmethyloxycarbonyl; Phe: phenylalanine; Tyr: tyrosine; p: phosphate group). This fibrilnano construction around the SiNPs@AP forms a uniform gel that, unpredictably, experiences a macroscopic shape alteration over time. The observed phenomenon of hydrogel aging is most probably because of phase separation, which generates a dense phase concentrated with SiNPs and nanostructures in the center of the vial, encircled by a dilute phase that still comprises SiNPs and tripeptide derivative. The hypothesis is that phase separation is not a syneresis process. This amendment is noticed when the enzymes are localized on the SiNPs. The dense phase shrinks over several days of storage until it reaches a persistent volume [146].

The presented examples of supramolecular gelation in the presence of enzymes or cells can be a very good alternative to using polymers and following crosslinking by harmful reagents, resulting in a loss of activity or viability. The drawback of the presented systems may be the high cost of production.

### 9.1. β-Glucosidase Immobilization

To preserve catalytic activity after the immobilization of partially purified β-glucosidase extract from *Aspergillus* sp., carriers and substrates were screened for the immobilization of the β-glucosidase enzyme preparation Cytolase PCL 5. PEI cellulose, α and γ aluminum oxide, and CHI may interact with 3-aminopropyltrimethoxysilane (APTS) carriers utilized for biomolecule-immobilization purposes, with a loading capacity of 1.3% and 18%. Chemical binding of β-glucosidase from *Pyrococcus Furiosus* in gelatin support and its zero valent crosslinking using transglutaminase yielded an inclusion degree ranging from 25 to 39%. In comparison, β-glucosidase obtained from almonds was immobilized with a yield loading capacity of only 5% [147].

*Cellulosimicrobium cellulans* KG3 was demonstrated, for which the particular activities of xanthanase and a secondary biocatalyst tangled in xanthan hydrolysis are as follows: xanthanase—19 U/g, β-glucosidase—3.4 ± 0.1 U/g, α-mannosidase—68.0 U/g, β-mannosidase—0.40 U/g, Endoglucanase—4 U/g, and xanthan lyase—2.2 U/mg. The biomolecules can complete xanthan hydrolysis for at least 40 cycles without losing activity or matrix denaturation [148].

Polyacrylamide cryogels of L-phenylalanine were prepared to support the adsorption and immobilization of β-glucosidase from *Thermoascus aurantiacus*, and the enzyme was obtained using the solid-state fermentation method. Next, adsorption was conducted, and the results were obtained at pH 3.0. Bioreactors immobilized with β-glucosidase showed reduction activity (%) ranging from 14.45 to 46 depending on the temperature and type of salt solution, and the ordinary encapsulated enzyme activity was 39.9 U/g of dry cryogel [149].

When the α-Glu enzyme was trapped in Dextran cryogels during synthesis, about 70% of the activity was lost. In contrast, Dextran cryogels with covalently immobilized α-Glu enzyme retained 91 ± 1.1% activity, could be used 10 times in a row, and were kept at 25 °C for 10 days, illustrating more than 50% of the native biocatalyst activity [150].

Alnadari et al. examined the immobilization of *Thermotoga maritima* β-glucosidase (Tm-β-Glu) on chitin, chitosan, and sodium alginate using magnetic Fe_3_O_4_ nanoparticles. It was possible to achieve β-glucosidase on chitin nanoparticles with 79% activity of the native state and produce 31.23% GOS after 10 cycles, demonstrating high efficiency and reusability [151].

Indian researchers Agrawal et al. immobilized β-glucosidase on SiO_2_ nanoparticles with a 52% efficiency and a 14% yield. The best parameters for utilization of this system were a temperature of 60 °C and a pH of 5. The immobilization resulted in the K_m_ value of β-glucosidase for the classic model reaction with p-nitrophenyl-β-d-glucopyranoside (pNPG) increasing from 0.9 to 1.07 mM and V max diminishing from 3.5 to 1.513 U/mg. The enzyme illustrated firmness and reuse up to 10 cycles with 70% in pNPG and 60% residual activity when utilized for the complex solution of sugarcane juice [152].

Sannino et al. analyzed the enzyme β-glucosidase (BG), which was fixed on wrinkled silica nanoparticles (WSN), and the procedure itself was conducted in anhydrous acetone under a stream of nitrogen. It was stated that BG had unusually high efficiency in acetone, changing 93.8% of cellobiose into glucose in just 20 min. The immobilized enzyme showed enhanced thermal stability relative to the free form and reserved 74.6% of native activity after incubation at 80 °C for 1 h. As expected, high temperatures lead to the acceleration of enzyme denaturation. Thus, the free solution of the enzyme retains only 57% of its activity after process treatment at 60 °C for 1 h and 47% after cultivation at 80 °C for 1 h [153].

Almulaiky et al. used AgNPs@TA-HMDA (acrylic textile coated with AgNP) and Ag(I)@TA-HMDA (acrylic textile coated with Ag ions) as substrates for the immobilization of β-glucosidase enzyme. The maximum encapsulation yield was reached with AgNPs@TA-HMDA at 92%, and Ag(I)@TA-HMDA reached 79.8%, causing an activity yield of 81% and 73%, respectively. At pH 6, the support of the immobilized enzyme was 4.6 g/kg, and at pH 8, it was 2.7 g/kg. The optimum temperature for the encapsulated enzyme was 60 °C, which was considerably higher than the optimum temperature of the native enzyme with a standard operation temperature of 50 °C [154].

Zeyadi et al. reviewed BG embedded in a CHI-MIL-Fe support, which had an immobilization load of 85% and recovered activity of 74%. The incorporated biocatalyst engaged 81% of its original activity after ten consecutive sequences and retained 69% of its native activity after 30 days of storage at 4 °C. Conversely, the native enzyme reserved 32% of the preliminary activity after a month. Some important kinetic parameters are as follows: Km was 13.4 and 6.98 mM for the encapsulated and free solution BG, respectively, and Vmax values were 3.96 and 1.72 U/mL, respectively [155].

Sometimes, covalent immobilization may lead to unexpected biocatalyst activity. β-glucosidase was overexpressed in crosslinked cryoPEI-al and PVA-al cells induced with isopropyl-b D-thiogalactopyranoside (IPTG), and subsequent analysis of b-glucosidase activity showed an increase in enzyme activity that reached almost 50% of the activity of crosslinked cells. CryoPEI-al and PVA-al with *E-coli* activity diminished faintly and, after 30 days, amounted to nearly 80% of the native enzyme activity [114].

The researchers gave an example of covalent immobilization: β-glucosidase from almond (Albgl) and *Thermotoga maritima* (Tmbgl) were first attached to oxidized and non-oxidized porous carbon cuboids (PCC) by crosslinking well-known reagents EDC and NHS, utilizing carbodiimide chemistry, which binds the -COOH and -OH groups of the nanomaterials to the primary amino functionalities of the biocatalyst. The immobilization yield of Albgl was as follows: PCC 72% (0.8 U mg of enzyme activity) and PCCox 90% (5 U/mg of enzyme activity); for covalent and non-covalent Albgl: PCC 80% (0.5 U/mg of enzyme activity) and PCCox 62% (9 U/mg of enzyme activity). For Tmbgl, the yield of covalent immobilization is PCC 95% (24 U/mg of enzyme activity) and PCCox 93% (35 U/mg of enzyme activity); non-covalent: PCC 94% (18 U/mg of enzyme activity) and PCCox 94% (37 U/mg of enzyme activity) [156].

Yaşar et al. used β-glucosidase purified via chromatography immobilized on superparamagnetic Fe^2+^/Fe^3+^ nanoparticles. The best temperature for native and immobilized BG was estimated as 55 °C and 37 °, and the ideal pH for native and immobilized BG is equal to 5.5. Thus, the uppermost activity was acquired when 22.9 μg of enzyme/25 mg of Fe^2+^ was immobilized with superparamagnetic nanoparticles. In 6 and 12 h tests, the immobilized enzymes caused a 43.56% reduction from the initial amount of oleuropein and induced a 100% reduction after 20 h. It was ascribed that the embedded enzyme retained its native activity at the end of six trials at 65% efficiency in hydrolyzing oleuropein [157].

Using β-glucosidase enzymes, cyanogenic glycosides, and plant glycosides, probiotics are produced from *Lactobaci llaceae* strains. Probiotic strains prevent pathogens and spoilage-causing germs in food matrices by manufacturing substances such as carbonic acids, bacteriocins, and amino acid metabolites [158].

### 9.2. Urease Immobilization

**Figure 8:**
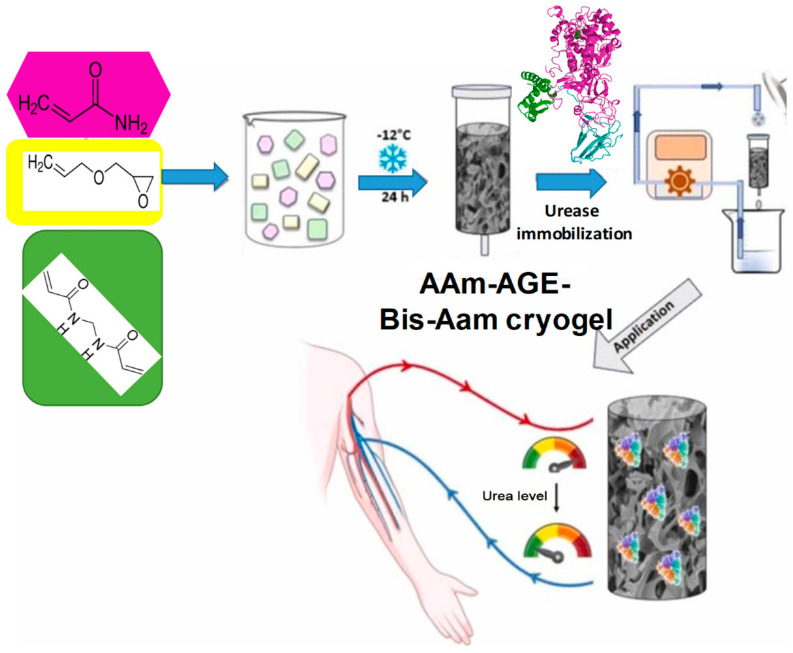
Urease immobilization with Poly(AAm-AGE)-based cryogels. Adapted with permission [159].

Poly(AAm-AGE)-based cryogels were fabricated using the covalent attachment of urease enzymes and used for the deposition of urea from human serum, the concept of which is presented in Figure 8. Immobilization was conducted using epoxy groups of allyl glycidyl ether, and the immobilization yield was 72 ± 2.65%. The urea removal rate from artificial human serum was 92%. Consequently, the immobilized urease can be reused up to 10 times [159].

**Figure 9:**
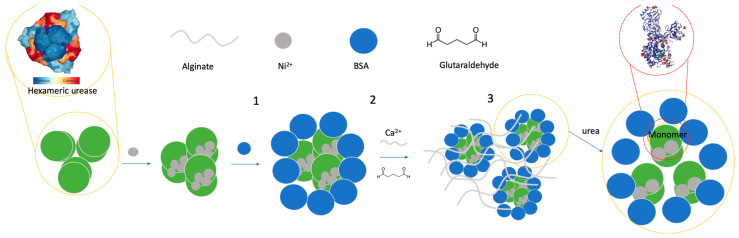
The urease-immobilization strategy. Adapted with permission [160].

Figure 9 represents the urease-incorporation approach wherein hexameric urease activity was enhanced by applying cofactor Ni^2+^ and additionally stabilized with albumin, followed by slight GA addition. This complex was included in alginate gel solidified with Ca^2+^ ions. As a result, in the presence of urea, the hexameric urease dissociated into active monomers, and the urease was additionally retained in the alginate, preventing the elution of the urease monomer. Urease in an alginate gel crosslinked with GA and Ca^2+^ reserved about 70% of its native activity, even after 2 weeks [160]. The PEI-modified eggshell membrane was used for urease immobilization using absorption and GA crosslinking methods. The surface roughness of the original membrane changed after PEI treatment and enzyme adsorption. The optimum temperature for all enzymes was 70 °C [161].

Porous silicon films obtained through electrochemical etching were used to immobilize urease via physical adsorption. The biocatalyst was exposed to different concentrations of urease in the native state and immobilized form, conducted using Cr^6+^, Cr^3+^, Cu^2+^, Fe^2+^, Cd^2+^, and Ni^2+^. The data indicate a higher sensitivity of native urease to trace amounts of urease Cr^3+^ compared to Cr^6+^. Judging from the IC_50_ values, the directive of inhibition of native urease was as follows: Ni^2+^ > Cu^2+^ > Cr^3+^ > Fe^2+^ > Cd^2+^ > Cr^6+^. For immobilized urease, Cr^3+^ and Cu^2+^ revealed significant inhibitory ability compared with all other tested metal ions [161].

Urease embedded inside a gel structure illustrated stability and enzymatic activity at room temperature (27–30 °C). The urease-coupled hydrogel was synthesized via an amino acid combination reaction. Urease-coupled hydrogel exhibited enzymatic efficiency at an alkaline pH and temperature below 60 °C. Urease-coupled hydrogel exhibited a substantial heightening of activity against thermal denaturation compared to the native enzyme [162].

This study used a biomimetic mineralization method to achieve urease immobilization in metal–organic frameworks (zeolithimidazolate framework-8, ZIF-8). Urease@ZIF-8 showed good recyclability during urea degradation, and it could retain 58.86% of the original activity after use for five cycles [163].

Fatma and Alatawi investigated urease immobilized on epichlorohydrin-crosslinked carboxymethylcellulose (ECH-CMC) beads, which were prepared via the graft copolymerization of polyacrylamide (PAm) on ECH-CMC beads. It is worth mentioning that after encapsulation, the ideal values were pH 8 and a temperature of 45 °C, indicating higher structural stability when immobilization was conducted. The kinetic parameters of Km and Vm were equal to 14 ± 0.7 mM and 2 ± 0.2 μmol NH_3_ min/mg of immobilized urease, respectively, and immobilized urease retained roughly 88% of its native biomolecule activity after the 10th reuse cycle [164].

The immobilization of covalent urease with high throughput was conducted by forming an amide bond between urease and amino-functionalized MNFs. The immobilization efficiency was about 95% while maintaining an initial urease-specific activity of 94.7%. The optimum pH for maximum activity of native and encapsulated urease was found to be 7 at 37 °C and 8 at 60 °C, respectively. The kinetic parameters exhibit that Km was 26 mM and 8 mM, and V max was 5.31 μmol/mg/min and 3.9 μmol/mg/min for native and immobilized protein structures, respectively [165].

The urease was efficiently immobilized on a new MOF called NEQC-340. The calculated immobilization efficiency was 90% while possessing about 87.7% of the native enzyme activity under the best conditions. The pre-eminent pH for these urease and NEQC-340@Urease is 7, and the optimal temperature equals 37 °C and 45 °C for native urease and NEQC-340@Urease, respectively. Moreover, 81% of the initial activity of NEQC-340@Urease was retained after 15 days of storage, and 55% of the activity was engaged after 10 repeated uses [166] (Table 4).

Nickel oxide polypyrrole (PPy) nanoparticles, namely, PPy-NiO, were placed on a Pt substrate, serving as a scaffold for immobilizing urease via physical adsorption. The invented electrode revealed a consistent and perfectly linear reply (the urea concentration ranged within 0.7–26.7 mM), extraordinary sensitivity (0.153 mA mM^−1^ cm^−2^), low detection limit (1.6 μM), long-term permanency up to 10 weeks, and prompt response time of 5 s. The established biosensor possessed high selectivity and was replicated using a PPy-NiO composite overloaded with encapsulated urease as urea biosensors [167].

He et al. developed urease-immobilized urea removal pellets, which were pre-produced via in situ crosslinking polymerization and phase-inversion methods. The results stated that these immobilized urease pellets showed a good urea decomposition capacity of 75 g/kg after incubation in an 80 mg/dL urea solution for 480 min, and the prepared pellets retained urea-removal activity [167].

In the studied article, micro/nanocellulose (MC)/NC) was magnetized with nano γ- Fe_2_O_3_ in nanoγ-Fe_2_O_3_@MC, oxidized to nanoγ-Fe_2_O_3_@MC to immobilize urease. The relative enzymatic efficiency of the immobilized enzyme was comparable to the native one (75–80% of the enzyme activity) [168].

Immobilizing cells, particularly by incorporating them into semipermeable polymer matrix structures, has several advantages over free cells. The leading advantages comprise a large cell density, improved specific productivity, simpler separation of products and biocatalysts, the ability to set up continuous bioreactors without cell washout, and biocatalyst reuse and cost reduction [169].

Polakovič et al. reported that the immobilization of whole cells with MAO-N led to a stable biocatalyst remaining active after 11 months of storing and unchanging up to 15 months after immobilization and continuous use. The production of *E. coli* expressing recombinant MAO-N was enlarged under controlled, pre-optimized settings (10% dissolved oxygen, pH 7). The amount of biomass was almost doubled compared to the flask culture [170].

*Escherichia coli* cells were mixed with N-cetyl-N,N,N,N-trimethylammonium bromide surfactant, and the immobilization technique was performed via chitosan 3% crosslinking using GA. The permeabilization of cells led to an increase in penicillin G acylase conversion by 9% within a quarter of an hour compared with intact *E.coli*. However, the immobilization on the chitosan matrix reduced conversion compared with non-immobilized treated *E. coli* (by 13%). The biocatalyst demonstrated acceptable operation stability, retaining more than 90% of the initial activity within 20 cycles. More than 200 days of storage in PBS at a temperature of 4 °C resulted in only 84% of the initial activity [171].

*Bacillus amyloliquefaciens* were isolated from dairy waste. They were immobilized in calcium alginate granules to produce antimicrobial protein. In the third cycle of use, the highest activity of 7300 U/mL was noticed, and before the fifth cycle of re-cultivation, productivity was about 97–94%. The seventh cycle of utilizing this immobilized microorganism retained 75.35% of the initial activity [172].

## 10. Conclusions

This review briefly describes isolation methods for microorganisms producing antibiotics, namely, bacteria (*Streptomyces anulatus*, *Bacillus megaterium*, *Bacillus subtilis*, *Bacillus lentus*, and *Lactococcus Lactis)* and fungi *(Aspergillus niger* (Coumarin and Griseofulvin), *Aspergillus fumigatus* (Coumarin), *Penicillium* sp. (penicillin), *Fusarium* sp. (fusidic acid), and *Streptomyces* sp. (tetracycline and Chlortetracycline)). Comprehensive immobilization techniques for cells and enzymes were discussed, and biocatalyst applications in various fields in the last decade were considered. For instance, examples of immobilization into hydrogels and cryogels of penicillinase from penicillin G acylase to produce antibiotic penicillin and *E. coli* and *Bacillus subtilis* bacterial cells are provided. High-activity enzyme-immobilization protocols (catalase, β-glucosidase, L-asparaginase, urease, etc.) are also presented. The processes of antibiotic production using fungi and bacteria producers are shown while considering their chemical structure and spectra of action. The data proving the efficiency of microorganism immobilization over conventional methods of suspension and biomass growth to obtain biological products such as antibiotics, enzymes, and precursors are summarized. The issue with natural polymers is their instability under some conditions and the release of degradation products that can contaminate the final product (technological impurity), leading to an additional purification step. The released compound may affect the enzymatic or metabolic activity of viable microorganisms; changing mechanical stability results in the loss of an immobilized enzyme or contamination of the substrate with cells, which, in turn, significantly influences the cost of the process. On the one side, synthetic polymers may be functionalized with highly reactive chemical groups, leading to significant loading of enzymes or whole bacteria on the carrier’s surface. On the other side, synthetic polymeric carriers have a carbon–carbon backbone, and therefore, these materials may be prepared with given mechanical properties and required porosity characteristics. There is no substrate/product diffusion restriction in the case of synthetic scaffolds due to the enzyme monolayer, while embedded biomolecules, mostly balk natural polysaccharides, have a disadvantage. Glutaraldehyde can be polymerized during storage; nevertheless, currently widely used gel-formation and bacteria- or enzyme-immobilization processes may negatively affect metabolic activity by inhibiting or altering the pathway, leading to the generation of unwanted compounds. Furthermore, Schiff’s bases are unstable in an acidic medium, which may trigger the release of GA into the medium. Ultimately, these issues require additional optimization of the purification process.

## Figures and Tables

**Table 2:** Fermentation of various antibiotics from Bactria genus *Bacillus*.

Producer	Antibiotic	Medium for Culturing	Product Yield
*Bacillus brevis*	Gramicidin-C	10% yeast hydrolysate	2000 µg/mL
*Bacillus brevis*	Gramicidin-C	Synthetic medium	38 g/L biomass, 5.28 g/L gramicidin C
*Bacillus subtilis*	Subtilosin A	Nutrient Agar M001, GRM agar, starch agar (Starch agar MRS)	activity was 102,400 AE/mL. 20 UU ml^−1^
*Bacillus pumillus*	Amicumacin A	SYC Culture Medium	50 mcg/mL

**Table 3:** Chemical nature and spectrum of action of antibiotics.

Antibiotic	Producer	Spectrum of Action
Fosfomycin	*Streptomyces fradiae*	Gram-positive microorganisms: *Enterococcus* spp., Gram-negative microorganisms: *Escherichia coli*, *Klebsiella* spp.
Neomycin	*Streptomyces fradiae*	*staphylococc*, *pneumococci* and Gram-negative bacteria
Actinomycin D	*Streptomyces chrysomallus*	Gram-positive bacteria, Gram-negative bacteria, cancer.
Endophenazine A	*Streptomyces anulatus*	Gram-positive bacteria, mycilial fungi, herbicidal activity against small cassava
Endophenazine B	*Streptomyces anulatus*	Gram-positive bacteria, mycilial fungi, herbicidal activity against small cassava
Epocarbazolin B	*Streptomyces anulatus*	5-lipoxygenase
Aldgamycin	*Streptomyces lavendulae*	*S. aureus*
Bacilysin	*Bacillus subtilis*	broad spectrum of bacteria, *Candida albicans.*
Subtilin	*Bacillus subtilis*	*Listeria monocytogenes*, *Candida* spp.
Bacillomycin D	*Bacillus subtilis*	various fungi, *M. globosa*
Rhizoctycin	*Bacillus subtilis*	various fungi, Rhizoctonia solani
Amicumacin A	*Bacillus pumillus*	*S. aureus*, *S. epidermidis*, *C. krusei*, *Cr. Neoformis*, *Prototheca* spp.
Gramicidin C	*Bacillus brevis*	*Streptococcus* spp.: *Streptococcus pneumoniae*, *Staphylococcus* spp., *anaerobic bacteria*
Tyrotricin	*Bacillus brevis*	Gram-positive microorganisms and spirochetes
Alvein	*Bacillus alvei*	*Mycobacterium tuberculosis*, *Bacillus sibiriae.*
Penicillinacylase	*Bacillus megaterium*	Gram-positive and Gram-negative bacteria.
Friulimicin	*Actinoplanes friuliensis*	staphylococci, pneumococci and Gram-negative bacteria
Penicillin G-acylases	*Escherichia coli*	Gram-positive and Gram-negative bacteria.
Tubermycin B	*Pseudomonas aeruginosa*	*A. flavus*, *C. albicans*, *T. rubrum*, fungi, *F. oxysporum*, *R. solani*

**Table 4:** Comparison of the results of immobilization and suspension.

Object	Immobilization on Agent	Activity after Immobilization	Activity in Suspension (%)	Number of Days (Cycles)
Bacteria				
*Escherichia coli* [75]	GA and Chitosan	90	13	20
*Escherichia coli* [114]	cryoPEI-al and PVA-al	80	60	30
*Escherichia coli* [112]	PVA hydrogel membranes (ECI-PVAHM)	91	45	40
*Escherichia coli* [115]	modified diatomite	86.2	74.1	40
*Bacillus amyloliquefaciens* [75]	calcium alginate beads	75.35	30	7
**Fungi**				
*Ganoderma lucidum* cells [117]	polyurethane foam	55.2	35.6	1
Fungal spores cells [118]	polymer	15	10	4
*Aspergillus oryzae cells* [120]	Allyl glycidyl ether monomer with acrylamide	97.37	68.2	20
*Rhizopus oryzae cells* [111]	PVA cryogel	94	52	20
*Fungi cells* [112]	PVA cryogels	91.5	50.6	80
**Enzyme**				
*Xylanase* [130]	PHG/PI-Xyl	69	49	30
*Trypsin* [131]	Cryogel diallyldimethylammonium chloride	80	71	20
*α-Glu enzyme* [150]	Dextran cryogels	50	30	10
L-asparaginase [133]	poly(HEMA-GMA) cryogel	54	30.7	28
Urease enzymes [159]	Poly(AAm-AGE)-based cryogels	71.9	-	10
β-glucosidase [147]	gelatin gel	39	5	5
β-glucosidase [152]	SiO_2_ nanoparticles	70	14.1	10
β-glucosidase [153]	wrinkled silica nanoparticles	74.6	43.1	1
β-glucosidase [155]	CS-MIL-Fe composite	81	32	30
β –glucosidase [114]	cryoPEI-al and PVA-al	80	50	30
Urease [166]	MOF called NEQC-340.	90	55	15

## Data Availability

Not applicable.
